# Slender salamanders (genus *Batrachoseps*) reveal Southern California to be a center for the diversification, persistence, and introduction of salamander lineages

**DOI:** 10.7717/peerj.9599

**Published:** 2020-08-14

**Authors:** Elizabeth L. Jockusch, Robert W. Hansen, Robert N. Fisher, David B. Wake

**Affiliations:** 1 Department of Ecology and Evolutionary Biology, University of Connecticut, Storrs, CT, USA; 2 Museum of Vertebrate Zoology, University of California, Berkeley, CA, United States of America; 3 Western Ecological Research Center, San Diego Field Station, San Diego, CA, U.S. Geological Survey, United States of America; 4 Department of Integrative Biology, University of California, Berkeley, CA, United States of America

**Keywords:** Phylogeography, Salamander, Introductions, Southern California, Biodiversity hotspot

## Abstract

**Background:**

The southern California biodiversity hotspot has had a complex geological history, with both plate tectonic forces and sea level changes repeatedly reconfiguring the region, and likely driving both lineage splittings and extinctions. Here we investigate patterns of genetic divergence in two species of slender salamanders (Plethodontidae: *Batrachoseps*) in this region. The complex geological history in combination with several organismal traits led us to predict that these species harbor multiple ancient mitochondrial lineages endemic to southern California. These species belong to a clade characterized by fine-scale mitochondrial structure, which has been shown to track ancient splits. Both focal species, *Batrachoseps major* and *B. nigriventris*, are relatively widely distributed in southern California, and estimated to have persisted there across millions of years. Recently several extralimital populations of *Batrachoseps* were found in the San Joaquin Valley of California, a former desert area that has been extensively modified for agriculture. The origins of these populations are unknown, but based on morphology, they are hypothesized to result from human-mediated introductions of *B. major*.

**Methods:**

We sequenced the mitochondrial gene *cytochrome b* from a geographically comprehensive sampling of the mitochondrial lineages of *B. major* and *B. nigriventris* that are endemic to southern California. We used phylogenetic analyses to characterize phylogeographic structure and identify mitochondrial contact zones. We also included the San Joaquin Valley samples to test whether they resulted from introductions. We used a bootstrap resampling approach to compare the strength of isolation-by-distance in both *Batrachoseps* species and four other salamander species with which they co-occur in southern California.

**Results:**

The northern lineage of *B. major* harbors at least eight deeply differentiated, geographically cohesive mitochondrial subclades. We identify geographic contact between many of these mtDNA lineages and some biogeographic features that are concordant with lineage boundaries. *Batrachoseps nigriventris* also has multiple deeply differentiated clades within the region. Comparative analyses highlight the smaller spatial scales over which mitochondrial divergence accumulates in *Batrachoseps* relative to most other salamander species in southern California. The extralimital populations of *Batrachoseps* from the San Joaquin Valley are assigned to *B. major* and are shown to result from at least two independent introductions from different source populations. We also suggest that *B. major* on Catalina Island, where it is considered native, may be the result of an introduction. Some of the same traits that facilitate the build-up of deep phylogeographic structure in *Batrachoseps* likely also contribute to its propensity for introductions, and we anticipate that additional introduced populations will be discovered.

## Introduction

The never-glaciated landscapes of western North America allow for a deep history of lineages within the region. Multiple processes, including seismic activity resulting from contact between the Pacific and North American plates, changes in sea level, riverine flooding, and climate change, have reconfigured the landscape (Nicholson et al., 1994; Engstrom, 1996; Miller, 2002; Gottscho, 2016). Range shifts and diversification among and within lineages have resulted, with regional species often showing significant genetic structuring with some degree of geographic concordance (Calsbeek, Thompson & Richardson, 2003; Lapointe & Rissler, 2005; Rissler et al., 2006; Vandergast et al., 2008; Gottscho, 2016). This geologically complex region is well known for genetic breaks in varied species including flightless arthropods (Bond, 2004; Bond et al., 2006; Vandergast et al., 2006; Vandergast et al., 2009; Chatzimanolis & Caterino, 2007; Polihronakis & Caterino, 2010), frogs (Phillipsen & Metcalf, 2009), fish (Richmond et al., 2015; Richmond et al., 2018), and lizards and snakes (Feldman & Spicer, 2006; Wood, Fisher & Reeder, 2008; Parham & Papenfuss, 2009; Leaché et al., 2009). This structuring is expected to be most extreme in lineages that arrived early in the region and have persisted with relatively low population connectivity.

**Figure 1:**
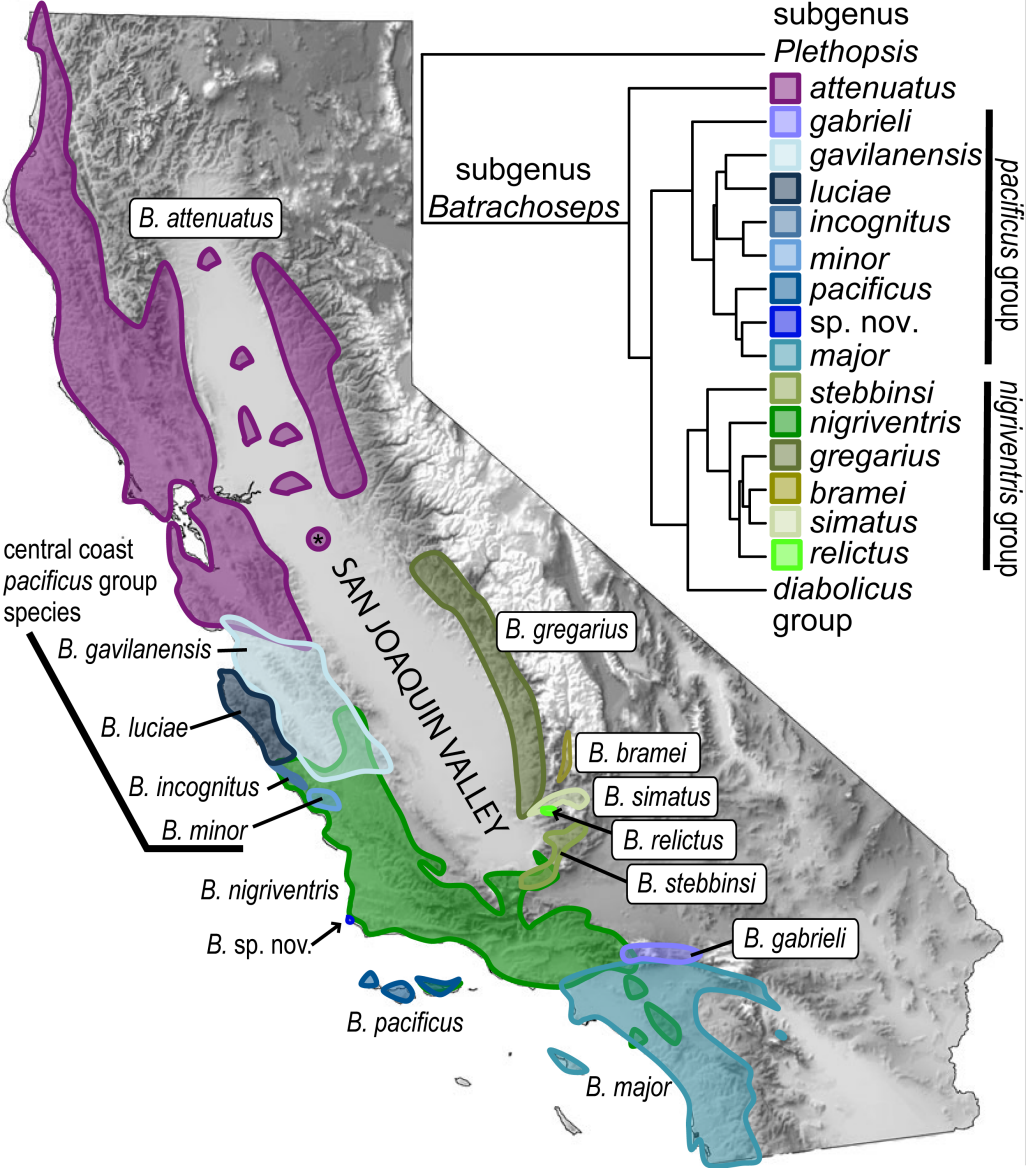
Overview of the genus *Batrachoseps*. California map (shaded by elevation) shows ranges of species in the *attenuatus*, *nigriventris* and *pacificus* species groups within the state; inset shows the species tree inferred from five nuclear genes. Asterisk indicates the Riverbank population of *B. attenuatus*, which may have been introduced. The map and tree are modified from Jockusch, Martínez-Solano & Timpe (2015).

Salamanders, especially those that are permanently terrestrial, offer striking examples for testing this hypothesis. Slender salamanders (genus *Batrachoseps*) are notable for their high degree of genetic structuring across geography (Yanev, 1978; Jockusch, Yanev & Wake, 2001; Jockusch & Wake, 2002; Martínez-Solano, Jockusch & Wake, 2007; Martínez-Solano et al., 2012), consistent with mark-recapture studies showing that movement is very low (2–30 m) over the course of a year (Hendrickson, 1954; Cunningham, 1960). The genus is distributed along the Pacific Coast and in the Sierra Nevada and adjoining inland mountain ranges of California, but largely absent from the Central Valley (Fig. 1). Once the genus was thought to consist of a single species spread across a vast California range (Hendrickson, 1954), but subsequent discoveries combined with modern molecular systematic analyses have shown that the genus has diverged into many species (Jockusch, Martínez-Solano & Timpe, 2015). Three species of slender salamanders (*B. gabrieli*, *B. major*, and *B. nigriventris*) have ancient mitochondrial lineages endemic to the southern California ecoregion (Wake & Jockusch, 2000; Jockusch & Wake, 2002; Martínez-Solano et al., 2012).

**Figure 2:**
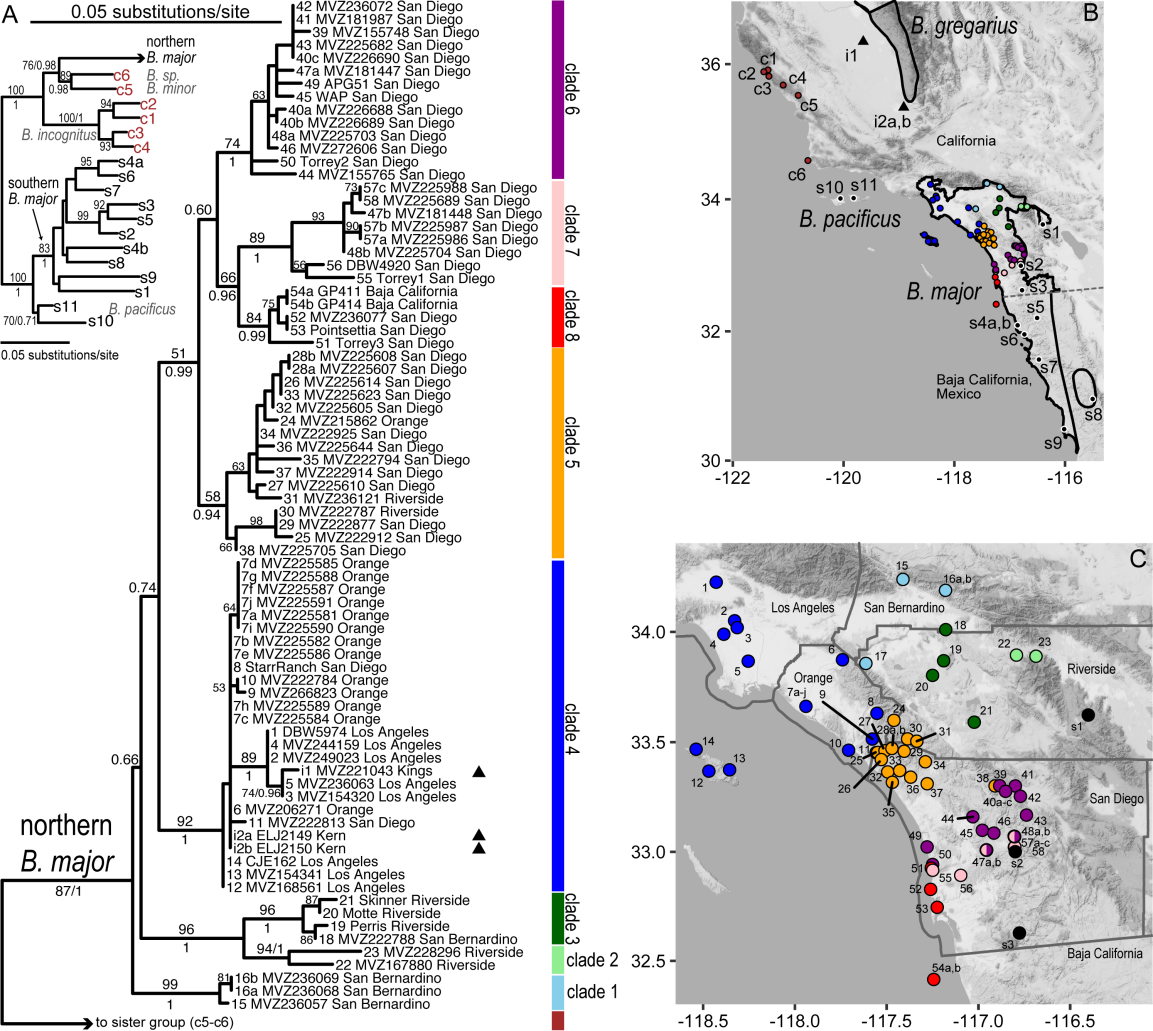
Phylogeographic structure of northern *B. major*. (A) Maximum likelihood tree for *B. major* (full outgroup sampling), with bootstrap values >50% shown on the tree for deeper nodes; values for shallower nodes have been removed to improve readability; Bayesian support values (posterior probabilities) are also shown (below or to the right) for the deepest nodes and nodes related to introduced samples. Sample names include population number, a letter if more than one individual was sampled from the population, museum or tissue voucher and county. Inset tree shows relationships of northern *B. major* to its closest mitochondrial relatives, with branch support values as in main tree; note the non-monophyly of *B. major* mtDNA. (B) Full geographic sampling for the *B. major* dataset. Range polygons are shown for *B. major* (unfilled) and *B. gregarius* (shaded), the species geographically closest to the San Joaquin Valley samples; range polygons are courtesy of the IUCN, with modifications to include all of our *B. major* samples. (C) Close-up of the southern California range of *B. major* showing the distribution of the 8 northern *B. major* clades discussed in the text. For A and B, triangles indicate San Joaquin Valley samples hypothesized to be introduced. Population numbers are as in Table 1; background map by Stamen Design, used under CC BY 3.0, with map data by OpenStreetMap, under ODbL. Color codes are matched in the map and tree: light blue-Clade 1; light green-Clade 2; dark green-Clade 3; royal blue-Clade 4; orange-Clade 5; purple-Clade 6; pink-Clade 7; red-Clade 8; black-southern *B. major* lineage (including *B. m. aridus*) and *B. pacificus*; brown-central coast.

The southernmost species is *Batrachoseps major*, the Southern California Slender Salamander, which has one of the largest geographic ranges of the 21 currently recognized species of *Batrachoseps* in terms of both area (IUCN, 2018) and linear extent. Its native range extends throughout more coastal regions of generally arid southern California, USA, and northern Baja California, Mexico, from the Los Angeles and San Bernardino basins to at least El Rosario on the Baja California Peninsula, with an isolated population in the Sierra San Pedro Mártir (Grismer, 2002; Martínez-Solano et al., 2012) (Fig. 2B). Perhaps more than any other species of *Batrachoseps*, *B. major* has successfully adapted to living in suburban environments (e.g., residential gardens) (Cunningham, 1960; Cornett, 1981; Hansen & Wake, 2005). The range of *B. major* overlaps with that of its congener *B. nigriventris*. Where they co-occur, the two species are generally separated by habitat, with *B. nigriventris* found primarily in upland oak woodland habitats and *B. major* widely distributed in lower elevation grassland habitats; microsympatry has been documented at ecotones (Lowe Jr & Zweifel, 1951). The range of *B. nigriventris* extends from the central Coast Ranges across the western and central Transverse Ranges and then south into the northern Peninsular Ranges (Fig. 1). The third species in the southern California ecoregion is *B. gabrieli*, which occurs in the central and eastern Transverse Ranges. Here we focus on genetic diversity in the first two species.

Our prior work with *B. major* and *B. nigriventris* identified a deeply diverged mitochondrial lineage in each that is endemic to the southern California ecoregion. In *B. major*, this is the northern lineage, encompassing most of the California portion of the range; this lineage is distributed across the Peninsular and eastern Transverse Ranges and extends into low basins that experienced repeated flooding through the Pleistocene (see Engstrom, 1996; Vandergast et al., 2006). The southern *B. major* mtDNA lineage extends from central San Diego Co. to the northwestern Baja California Peninsula. These two lineages within *B. major* are estimated to have diverged 10.1 mya, with diversification in the northern lineage beginning 3.2 mya (Martínez-Solano et al., 2012). The southern lineage of *B. nigriventris* is endemic to the southern California ecoregion and ranges from the southern flanks of the central Transverse Ranges south into the Peninsular Ranges; allozyme divergences are as high as D_Nei_ = 0.20, indicating a long-term presence (Wake & Jockusch, 2000). This lineage also occurs on one of the northern Channel Islands, which is geologically affiliated with the Transverse Ranges (Miller, 2002). The northern *B. nigriventris* mtDNA lineage occupies the western Transverse Ranges and the central Coast Ranges.

An indication of the complex history of both species is their non-monophyletic DNA. In both species, the southern California mtDNA lineage (i.e., northern *B. major* and southern *B. nigriventris*) is more closely related to mtDNA from other species outside the ecoregion than it is to conspecific mtDNA from the rest of the range (Wake & Jockusch, 2000; Jockusch & Wake, 2002). However, the status of each as a single species is consistent with allozymic and nuclear sequence data, which strongly support closer relationships between the conspecific pairs of mitochondrial lineages (Fig. 1), which are continuously distributed, than between the mtDNA lineage endemic to southern California and its geographically disjunct mitochondrial relatives (Yanev, 1978; Yanev, 1980; Wake & Jockusch, 2000; Martínez-Solano et al., 2012; Jockusch, Martínez-Solano & Timpe, 2015). This biogeographic pattern indicates that the mitochondrial lineages likely result from ancient breaks that have persisted in the face of geographic contact, rather than from recent introgression from another source (Wake & Jockusch, 2000; Jockusch & Wake, 2002; Wake, 2006).

The southern California ecoregion is separated from the Central Valley by the Transverse Ranges, which rotated from the typical north-south orientation into their current east–west orientation across the Miocene and Pliocene (Nicholson et al., 1994; Miller, 2002; Gottscho, 2016). Although largely absent from the Central Valley, *Batrachoseps* has been found in isolated pockets there (Fig. 1). Most of these are in northern California, belong to the species *B. attenuatus*, and are thought to have persisted since the last glacial maximum, when the Central Valley was cooler and wetter. During glacial periods, several salamander lineages, including *B. attenuatus*, dispersed across the Valley from the Coast Ranges to the Sierra Nevada foothills (Stebbins, 1957; Martínez-Solano, Jockusch & Wake, 2007; Reilly, Corl & Wake, 2015). Surprisingly, two populations of *Batrachoseps* were recently found on the floor of the southern San Joaquin Valley (the southern portion of California’s Great Central Valley), a hot and dry region that is generally inhospitable for salamanders. Both sites were located in residential neighborhoods, with yards currently or formerly landscaped with non-native (i.e., nursery-propagated) plants that receive watering during the dry season. Each of these sites contained a mix of adults and younger individuals, suggesting reproducing populations. These populations are outside the known range of any species of *Batrachoseps*, and their morphology suggested that they could be *B. major*, raising the possibility that they are introduced. Introductions of herpetofauna are a growing problem, although the phenomenon is much more frequently documented in frogs than salamanders (Kraus, 2009).

In this study, we used expanded sampling of individuals from the northern *B. major* and southern *B. nigriventris* mtDNA lineages to address four questions. First, do these lineages display deep mitochondrial structure in the southern California ecoregion, as we would predict for low vagility species with a long history in the region? Second, how does the extent and depth of fine-scale geographic structure in *Batrachoseps* from southern California compare to that in other salamanders with which these species co-occur? Third, are the populations of *Batrachoseps* from the San Joaquin Valley introduced, and if so, where did they originate? The fine-scale mitochondrial structure characteristic of *Batrachoseps* makes it possible to pinpoint origins of introduced populations relatively precisely. Fourth, do the data give evidence of other introductions? These questions about phylogeography and introductions are connected because both are, to a substantial degree, questions about the ability of a lineage to persist in a particular place and we discuss traits of *Batrachoseps* that likely enable long-term persistence.

**Table 1:** Voucher and locality information for samples included in the *B. major* dataset. The number sign (#) is population number, also used in Figs. 2, 6 and Fig. S3); letters at end designate multiple individuals from the same sampling locality; letters at beginning designate introduced (i), southern (s) and central coast (c) populations; SpecimenID is museum voucher when available (MVZ = Museum of Vertebrate Zoology Herpetology Collection, University of California, Berkeley; IBH: Instituto de Biología, National Autonomous University of Mexico) or collector (AP, CJE, DBW, ELJ, GP) or other designation for samples that have not been accessioned or lack specimen vouchers; * indicates samples hypothesized to be introduced; subclades are numbered within northern *B. major*; S = southern *B. major* or *B. pacificus*; CC = central coast samples nested within *B. major*.

**#**	**SpecimenID**	**Species**	**Clade**	**Locality**	**Lat (°N)**	**Long (°W)**
1	DBW5974	*B. major*	4	Los Angeles Co., CA, USA	34.2233	−118.4300
2	MVZ249023	*B. major*	4	Los Angeles Co., CA, USA	34.0492	−118.3297
3	MVZ154320	*B. major*	4	Los Angeles Co., CA, USA	34.0184	−118.3155
4	MVZ244159	*B. major*	4	Los Angeles Co., CA, USA	33.9885	−118.3886
5	MVZ236063	*B. major*	4	Los Angeles Co., CA, USA	33.8666	−118.2555
6	MVZ206271	*B. major*	4	Orange Co., CA, USA	33.8729	−117.7417
7a	MVZ225581	*B. major*	4	Orange Co., CA, USA	33.6619	−117.9410
7b	MVZ225582	*B. major*	4	Orange Co., CA, USA	33.6619	−117.9410
7c	MVZ225584	*B. major*	4	Orange Co., CA, USA	33.6619	−117.9410
7d	MVZ225585	*B. major*	4	Orange Co., CA, USA	33.6619	−117.9410
7e	MVZ225586	*B. major*	4	Orange Co., CA, USA	33.6619	−117.9410
7f	MVZ225587	*B. major*	4	Orange Co., CA, USA	33.6619	−117.9410
7g	MVZ225588	*B. major*	4	Orange Co., CA, USA	33.6619	−117.9410
7h	MVZ225589	*B. major*	4	Orange Co., CA, USA	33.6619	−117.9410
7i	MVZ225590	*B. major*	4	Orange Co., CA, USA	33.6619	−117.9410
7j	MVZ225591	*B. major*	4	Orange Co., CA, USA	33.6619	−117.9410
8	StarrRanch	*B. major*	4	Orange Co., CA, USA	33.6303	−117.5539
9	MVZ266823	*B. major*	4	Orange Co., CA, USA	33.5134	−117.5790
10	MVZ222784	*B. major*	4	Orange Co., CA, USA	33.4625	−117.7083
11	MVZ222813	*B. major*	4	San Diego Co., CA, USA	33.4517	−117.5586
12	MVZ168561	*B. major*	4	Los Angeles Co., CA, USA	33.3675	−118.4701
13	MVZ154341	*B. major*	4	Los Angeles Co., CA, USA	33.3399	−118.3294
14	CJE162	*B. major*	4	Los Angeles Co., CA, USA	33.4670	−118.5395
15	MVZ236057	*B. major*	1	San Bernardino Co., CA, USA	34.2360	−117.4119
16a	MVZ236068	*B. major*	1	San Bernardino Co., CA, USA	34.1867	−117.1800
16b	MVZ236069	*B. major*	1	San Bernardino Co., CA, USA	34.1867	−117.1800
17	MVZ167815	*B. major*	1	Riverside Co., CA, USA	33.8565	−117.6141
18	MVZ222788	*B. major*	3	San Bernardino Co., CA, USA	34.0093	−117.1788
19	Perris	*B. major*	3	Riverside Co., CA, USA	33.8686	−117.1902
20	Motte	*B. major*	3	Riverside Co., CA, USA	33.8023	−117.2506
21	Skinner	*B. major*	3	Riverside Co., CA, USA	33.5899	−117.0233
22	MVZ167880	*B. major*	2	Riverside Co., CA, USA	33.8941	−116.7932
23	MVZ228296	*B. major*	2	Riverside Co., CA, USA	33.8889	−116.6869
24	MVZ215862	*B. major*	5	Orange Co., CA, USA	33.5983	−117.4612
25	MVZ222912	*B. major*	5	San Diego Co., CA, USA	33.4524	−117.5499
26	MVZ225614	*B. major*	5	San Diego Co., CA, USA	33.4200	−117.5300
27	MVZ225610	*B. major*	5	San Diego Co., CA, USA	33.4600	−117.5100
28a	MVZ225607	*B. major*	5	San Diego Co., CA, USA	33.4705	−117.4722
28b	MVZ225608	*B. major*	5	San Diego Co., CA, USA	33.4705	−117.4722
29	MVZ222877	*B. major*	5	San Diego Co., CA, USA	33.4578	−117.4053
30	MVZ222787	*B. major*	5	Riverside Co., CA, USA	33.5141	−117.3868
31	MVZ236121	*B. major*	5	Riverside Co., CA, USA	33.5037	−117.3365
32	MVZ225605	*B. major*	5	San Diego Co., CA, USA	33.3638	−117.4951
33	MVZ225623	*B. major*	5	San Diego Co., CA, USA	33.3700	−117.4300
34	MVZ222925	*B. major*	5	San Diego Co., CA, USA	33.4100	−117.2900
35	MVZ222794	*B. major*	5	San Diego Co., CA, USA	33.3167	−117.4697
36	MVZ225644	*B. major*	5	San Diego Co., CA, USA	33.3400	−117.3700
37	MVZ222914	*B. major*	5	San Diego Co., CA, USA	33.3100	−117.2800
38	MVZ225705	*B. major*	5	San Diego Co., CA, USA	33.3000	−116.9100
39	MVZ155748	*B. major*	6	San Diego Co., CA, USA	33.3011	−116.8849
40a	MVZ226688	*B. major*	6	San Diego Co., CA, USA	33.2754	−116.8527
40b	MVZ226689	*B. major*	6	San Diego Co., CA, USA	33.2754	−116.8527
40c	MVZ226690	*B. major*	6	San Diego Co., CA, USA	33.2754	−116.8527
41	MVZ181987	*B. major*	6	San Diego Co., CA, USA	33.2999	−116.7994
42	MVZ236072	*B. major*	6	San Diego Co., CA, USA	33.2528	−116.7717
43	MVZ225682	*B. major*	6	San Diego Co., CA, USA	33.1670	−116.7387
44	MVZ155765	*B. major*	6	San Diego Co., CA, USA	33.1592	−117.0311
45	WAP	*B. major*	6	San Diego Co., CA, USA	33.0976	−116.9795
46	MVZ272606	*B. major*	6	San Diego Co., CA, USA	33.0848	−116.9164
47a	MVZ181447	*B. major*	6	San Diego Co., CA, USA	33.0082	−116.9569
47b	MVZ181448	*B. major*	7	San Diego Co., CA, USA	33.0082	−116.9569
48a	MVZ225703	*B. major*	6	San Diego Co., CA, USA	33.0705	−116.8060
48b	MVZ225704	*B. major*	7	San Diego Co., CA, USA	33.0705	−116.8060
49	APG51	*B. major*	6	San Diego Co., CA, USA	33.0217	−117.2805
50	Torrey2	*B. major*	6	San Diego Co., CA, USA	32.9414	−117.2505
51	Torrey3	*B. major*	8	San Diego Co., CA, USA	32.9250	−117.2574
52	MVZ236077	*B. major*	8	San Diego Co., CA, USA	32.8275	−117.2612
53	Pointsettia	*B. major*	8	San Diego Co., CA, USA	32.7452	−117.2252
54a	GP411	*B. major*	8	Baja California, Mexico	32.4139	−117.2442
54b	GP414	*B. major*	8	Baja California, Mexico	32.4139	−117.2442
55	Torrey1	*B. major*	7	San Diego Co., CA, USA	32.9149	−117.2500
56	DBW4920	*B. major*	7	San Diego Co., CA, USA	32.8918	−117.0971
57a	MVZ225986	*B. major*	7	San Diego Co., CA, USA	33.0219	−116.8022
57b	MVZ225987	*B. major*	7	San Diego Co., CA, USA	33.0219	−116.8022
57c	MVZ225988	*B. major*	7	San Diego Co., CA, USA	33.0219	−116.8022
58	MVZ225689	*B. major*	7	San Diego Co., CA, USA	33.0202	−116.8047
c1	ELJ2052	*B. incognitus*	CC	Monterey Co., CA, USA	35.9138	−121.3639
c2	MVZ266756	*B. incognitus*	CC	Monterey Co., CA, USA	35.8879	−121.4405
c3	MVZ266750	*B. incognitus*	CC	Monterey Co., CA, USA	35.8219	−121.3478
c4	MVZ224790	*B. incognitus*	CC	San Luis Obispo Co., CA, USA	35.6931	−121.0909
c5	MVZ237245	*B. minor*	CC	San Luis Obispo Co., CA, USA	35.5419	−120.8180
c6	ELJ1775	*B.* sp.	CC	Santa Barbara Co., CA, USA	34.5783	−120.6436
i1	MVZ221043*	*B. major*	4	Kings Co., CA, USA	36.3308	−119.6500
i2a	ELJ2149*	*B. major*	4	Kern Co., CA, USA	35.3655	−118.9129
i2b	ELJ2150*	*B. major*	4	Kern Co., CA, USA	35.3655	−118.9129
s1	MVZ222553	*B. m. aridus*	S	Riverside Co., CA, USA	33.6225	−116.4018
s2	MVZ225684	*B. major*	S	San Diego Co., CA, USA	32.9997	−116.7998
s3	Marron	*B. major*	S	San Diego Co., CA, USA	32.6272	−116.7770
s4a	Mision1	*B. major*	S	Baja California, Mexico	32.0930	−116.8571
s4b	Mision3	*B. major*	S	Baja California, Mexico	32.0930	−116.8571
s5	APG72	*B. major*	S	Baja California, Mexico	32.2046	−116.5044
s6	IBH14152	*B. major*	S	Baja California, Mexico	31.9536	−116.7350
s7	APG36	*B. major*	S	Baja California, Mexico	31.5672	−116.4720
s8	DBW6026	*B. major*	S	Baja California, Mexico	30.9582	−115.5020
s9	APG50	*B. major*	S	Baja California, Mexico	30.4900	−116.0158
s10	MVZ232916	*B. pacificus*	S	Santa Barbara Co., CA, USA	34.0121	−120.0562
s11	MVZ156098	*B. pacificus*	S	Santa Barbara Co., CA, USA	34.0178	−119.8183

## Materials & Methods

### Population sampling for *B. major*

*B. major* was sampled from throughout its range (Table 1, Fig. 2). We focused on the clade previously identified as northern *B. major*, because it is endemic to the California ecoregion region and, in contrast to southern *B. major*, has not been subject to intensive sampling in prior work (e.g., Martínez-Solano et al., 2012). The final data matrix includes 75 individuals from 58 localities in the northern mitochondrial lineage. Twenty-six are from our prior studies (Wake & Jockusch, 2000; Jockusch & Wake, 2002; Martínez-Solano et al., 2012; Jockusch, Martínez-Solano & Timpe, 2015) (GenBank accession numbers JQ250195–JQ250218); the remaining 49 are newly reported here and have been deposited in GenBank (accession numbers MN736845; MN736847 –MN736849; MN736852 –MN736898). Most localities were represented by 1 or 2 individuals; 10 individuals were sequenced from one locality (population 7). We emphasized sampling discrete localities over many individuals per locality because studies of *Batrachoseps* consistently find that mitochondrial haplotypes have very small geographic ranges, while variation within populations is relatively limited in comparison (Martínez-Solano, Jockusch & Wake, 2007; Martínez-Solano & Lawson, 2009; Jockusch et al., 2012). Work involving live vertebrates was approved by the Institutional Animal Care and Use Committee of the University of Connecticut (protocols A18-003, A15-002, A11-002, A08-009, and A04-213). Field sampling was conducted under scientific collecting permits (SC-838, SC-8535, SC-013377) issued by the California Department of Fish and Wildlife. Additional permits for this work were obtained from the following entities: California Department of Parks and Recreation, National Audubon Society, Riverside County Parks, University of California Natural Reserve System, Zoological Society of San Diego and Environmental Security Office of the Marine Corps Base Camp Pendleton.

The sampling also included representatives of two populations of *Batrachoseps* from the San Joaquin Valley (*N* = 3; GenBank MN736846, MN736850 –MN736851; Fig. 2B). In February 1993, one of us (RWH) was informed of the presence of *Batrachoseps* within the city limits of Hanford, Kings County (population i1; 36.330946, −119.649975), and subsequently three specimens (MVZ 221043–221045) tentatively identified as *B. major* were collected. Twenty-three years later (April 2015), three salamanders morphologically consistent with *B. major* were collected in Bakersfield, Kern County (population i2; 35.365520, −118.912936; ELJ2149–2151).

To ensure that the mitochondrial diversity of *B. major* was fully represented, we also selected 10 individuals from our prior work that span the diversity of the southern *B. major* mtDNA lineage, including *B. m. aridus*, and eight individuals assigned to the four other taxa (*B. pacificus*, *B. minor*, *B. incognitus*, and *B.* sp. nov. from Santa Barbara Co.) that are descended from the most recent common ancestor of northern + southern *B. major* mtDNA (Jockusch & Wake, 2002; Martínez-Solano et al., 2012; Jockusch, Martínez-Solano & Timpe, 2015).

### Population sampling for *B. nigriventris*

**Figure 3:**
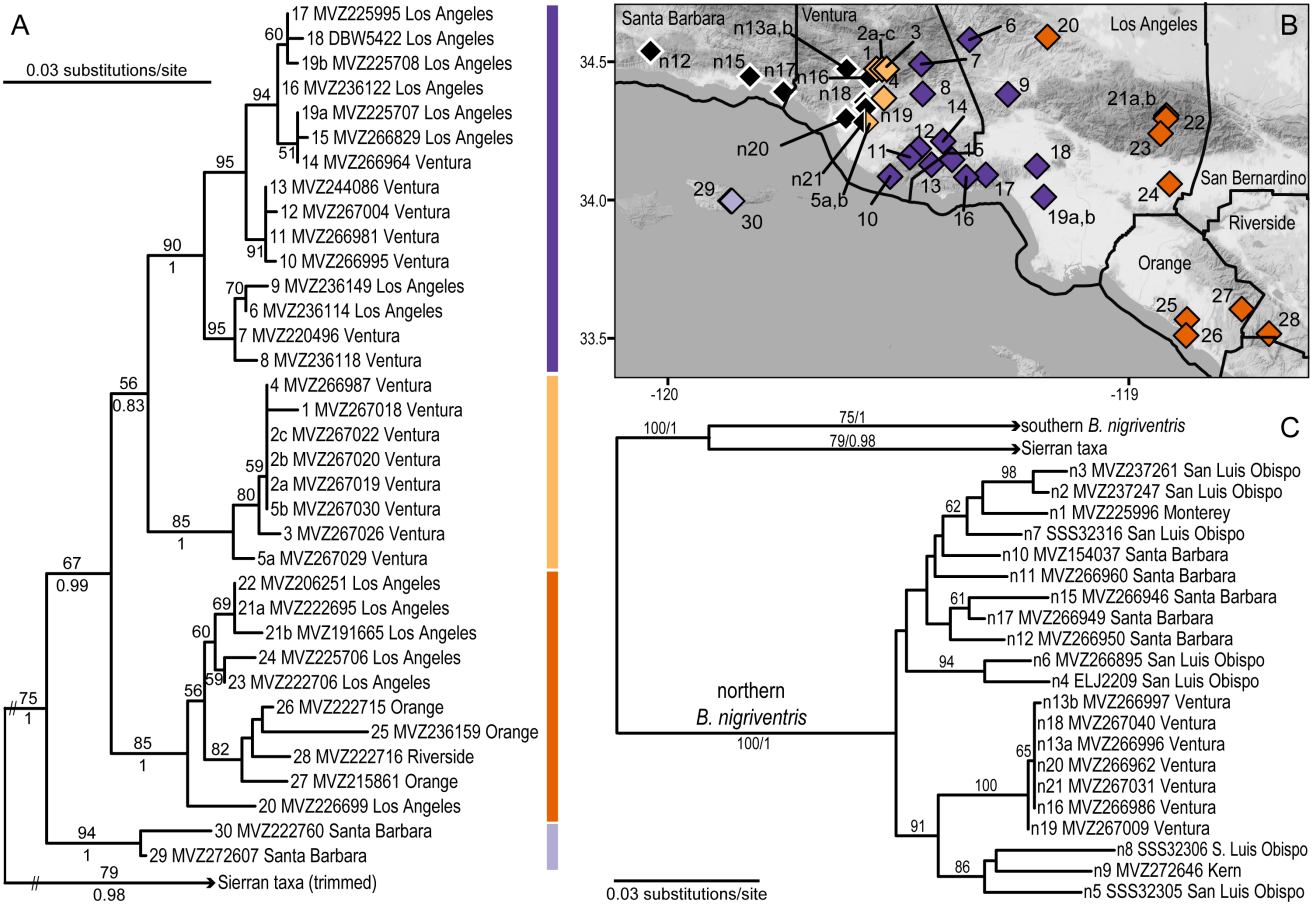
Phylogeographic structure of southern *B. nigriventris*. (A) Maximum likelihood tree for the southern mitochondrial lineage within *B. nigriventris*. Bootstrap values above 50% are shown on the tree; posterior probabilities are included for deeper nodes. Samples are named by population number (followed by letter if more than one sample), museum voucher, and county. (B) There are four deeply differentiated clades, geographically cohesive within southern *B. nigriventris*, the island clade (light purple) and three mainland clades (light orange, dark purple, and dark orange, for the western, central and eastern clades, respectively); northern *B. nigriventris* is shown in black; some northern *B. nigriventris* and all Sierran samples are outside the region shown on the map. Background map by Stamen Design, used under CC BY 3.0, with map data by OpenStreetMap, under ODbL. (C) Maximum likelihood tree for outgroups to the southern mitochondrial lineage within *B. nigriventris*, labeled as in A; note the non-monophyly of *B. nigriventris* mtDNA. Population numbers are as in Table 2.

The *B. nigriventris* dataset includes 63 individuals, with sampling focused on the southern lineage and its contact zone with the northern lineage (Table 2, Fig. 3). Of these, 35 (23 new in this study) are assigned to the southern *B. nigriventris* lineage, 21 (17 new) to the northern *B. nigriventris* lineage; and 7 (0 new) to other species in the *B. nigriventris* group (*B. bramei*, *B. simatus*, and *B. relictus*) that carry mtDNA descended from the most recent common ancestor of northern + southern *B. nigriventris* mtDNA. For the northern lineage, 10 (all new) were from the vicinity of the mitochondrial contact zone, while the other 11 were selected to represent the additional mitochondrial diversity present elsewhere in the northern lineage. The published data for *B. nigriventris* come from our earlier studies (Wake & Jockusch, 2000; Jockusch & Wake, 2002; Jockusch et al., 2012; Jockusch, Martínez-Solano & Timpe, 2015); sequences that were not already available in GenBank have been deposited under accession numbers MN736899 –MN736949.

### DNA sequencing and phylogenetic analysis

Because of its high rate of evolution (Mueller, 2006) and evidence that mtDNA tracks ancient breaks in *Batrachoseps* (Jockusch & Wake, 2002; Wake, 2006; Martínez-Solano, Jockusch & Wake, 2007; Martínez-Solano et al., 2012), we used the mitochondrial gene *cytochrome b* (*cytb*) to characterize diversity within *B. major* and *B. nigriventris* and to pinpoint the origin of the San Joaquin Valley samples. The targeted fragment is 784 bp corresponding to positions 21–804 of the *cytb* gene from the *B. nigriventris* mitochondrial genome (GenBank Accessions NC_028184.1) and is flanked by the primers MVZ15 and MVZ16 (Moritz, Schneider & Wake, 1992). Because data were collected over an extended period, wet lab methods followed an evolving set of protocols, which have been described in our previous work (Jockusch & Wake, 2002; Martínez-Solano et al., 2012; Jockusch, Martínez-Solano & Timpe, 2015). The earliest sequences were generated using radiolabeled nucleotides on slab gels, with the results read by eye, and the most recent via fluorescently-labeled nucleotides separated on an ABI 3130 Genetic Analyzer, with subsequent checking of the chromatograms in Sequencher v. 5.1 (Gene Codes Corporation).

**Table 2:** Voucher and locality information for samples included in the *B. nigriventris* dataset. Columns as in Table 1; # is population number also used in Figs. 3, 6 and Fig. S4; clade designations are as follows: island = island, m-E, m-c and m-W = mainland East, central and West clades within southern *B. nigriventris*; N = northern *B. nigriventris*; Sierran = Sierran samples nested within *B. nigriventris*.

**#**	**Specimen ID**	**Species**	**Clade**	**Locality**	**Lat (^∘^N)**	**Long (^∘^W)**
1	MVZ267018	*B. nigriventris*	m-E	Ventura Co., CA, USA	34.4291	−119.0908
2a	MVZ267019	*B. nigriventris*	m-E	Ventura Co., CA, USA	34.4395	−119.0808
2b	MVZ267020	*B. nigriventris*	m-E	Ventura Co., CA, USA	34.4395	−119.0808
2c	MVZ267022	*B. nigriventris*	m-E	Ventura Co., CA, USA	34.4395	−119.0808
3	MVZ267026	*B. nigriventris*	m-E	Ventura Co., CA, USA	34.4427	−119.0769
4	MVZ266987	*B. nigriventris*	m-E	Ventura Co., CA, USA	34.3650	−119.0622
5a	MVZ267029	*B. nigriventris*	m-E	Ventura Co., CA, USA	34.2790	−119.1406
5b	MVZ267030	*B. nigriventris*	m-E	Ventura Co., CA, USA	34.2790	−119.1406
6	MVZ236114	*B. nigriventris*	m-c	Los Angeles Co., CA, USA	34.5742	−118.6908
7	MVZ220496	*B. nigriventris*	m-c	Ventura Co., CA, USA	34.4869	−118.9001
8	MVZ236118	*B. nigriventris*	m-c	Ventura Co., CA, USA	34.3803	−118.8942
9	MVZ236149	*B. nigriventris*	m-c	Los Angeles Co., CA, USA	34.3789	−118.5244
10	MVZ266995	*B. nigriventris*	m-c	Ventura Co., CA, USA	34.0843	−119.0356
11	MVZ266981	*B. nigriventris*	m-c	Ventura Co., CA, USA	34.1537	−118.9500
12	MVZ267004	*B. nigriventris*	m-c	Ventura Co., CA, USA	34.1842	−118.9107
13	MVZ244086	*B. nigriventris*	m-c	Ventura Co., CA, USA	34.1267	−118.8557
14	MVZ266964	*B. nigriventris*	m-c	Ventura Co., CA, USA	34.2107	−118.8051
15	MVZ266829	*B. nigriventris*	m-c	Los Angeles Co., CA, USA	34.1444	−118.7662
16	MVZ236122	*B. nigriventris*	m-c	Los Angeles Co., CA, USA	34.0820	−118.7051
17	MVZ225995	*B. nigriventris*	m-c	Los Angeles Co., CA, USA	34.0900	−118.6200
18	DBW5422	*B. nigriventris*	m-c	Los Angeles Co., CA, USA	34.1214	−118.3981
19a	MVZ225707	*B. nigriventris*	m-c	Los Angeles Co., CA, USA	34.0105	−118.3683
19b	MVZ225708	*B. nigriventris*	m-c	Los Angeles Co., CA, USA	34.0105	−118.3683
20	MVZ226699	*B. nigriventris*	m-W	Los Angeles Co., CA, USA	34.5836	−118.3526
21a	MVZ222695	*B. nigriventris*	m-W	Los Angeles Co., CA, USA	34.2923	−117.8390
21b	MVZ191665	*B. nigriventris*	m-W	Los Angeles Co., CA, USA	34.2915	−117.8384
22	MVZ206251	*B. nigriventris*	m-W	Los Angeles Co., CA, USA	34.3029	−117.8366
23	MVZ222706	*B. nigriventris*	m-W	Los Angeles Co., CA, USA	34.2382	−117.8618
24	MVZ225706	*B. nigriventris*	m-W	Los Angeles Co., CA, USA	34.0578	−117.8223
25	MVZ236159	*B. nigriventris*	m-W	Orange Co., CA, USA	33.5692	−117.7473
26	MVZ222715	*B. nigriventris*	m-W	Orange Co., CA, USA	33.5116	−117.7509
27	MVZ215861	*B. nigriventris*	m-W	Orange Co., CA, USA	33.6066	−117.5094
28	MVZ222716	*B. nigriventris*	m-W	Riverside Co., CA, USA	33.5180	−117.3914
29	MVZ272607	*B. nigriventris*	island	Santa Barbara Co., CA, USA	33.9966	−119.7276
30	MVZ222760	*B. nigriventris*	island	Santa Barbara Co., CA, USA	33.9962	−119.7213
n1	MVZ225996	*B. nigriventris*	N	Monterey Co., CA, USA	35.8371	−121.3902
n2	MVZ237247	*B. nigriventris*	N	San Luis Obispo Co., CA, USA	35.5419	−120.8180
n3	MVZ237261	*B. nigriventris*	N	San Luis Obispo Co., CA, USA	35.4760	−120.8452
n4	ELJ2209	*B. nigriventris*	N	San Luis Obispo Co., CA, USA	35.3937	−120.4657
n5	SSS32305	*B. nigriventris*	N	San Luis Obispo Co., CA, USA	35.2617	−119.9403
n6	MVZ266895	*B. nigriventris*	N	San Luis Obispo Co., CA, USA	35.1945	−120.4538
n7	SSS32316	*B. nigriventris*	N	San Luis Obispo Co., CA, USA	35.1755	−120.7813
n8	SSS32306	*B. nigriventris*	N	San Luis Obispo Co., CA, USA	35.0778	−119.7737
n9	MVZ272646	*B. nigriventris*	N	Kern Co., CA, USA	34.9841	−119.1862
n10	MVZ154037	*B. nigriventris*	N	Santa Barbara Co., CA, USA	34.7947	−120.0454
n11	MVZ266960	*B. nigriventris*	N	Santa Barbara Co., CA, USA	34.5839	−120.5603
n12	MVZ266950	*B. nigriventris*	N	Santa Barbara Co., CA, USA	34.5334	−120.0749
n13a	MVZ266996	*B. nigriventris*	N	Ventura Co., CA, USA	34.4693	−119.2239
n13b	MVZ266997	*B. nigriventris*	N	Ventura Co., CA, USA	34.4693	−119.2239
n15	MVZ266946	*B. nigriventris*	N	Santa Barbara Co., CA, USA	34.4426	−119.6435
n16	MVZ266986	*B. nigriventris*	N	Ventura Co., CA, USA	34.4384	−119.1245
n17	MVZ266949	*B. nigriventris*	N	Santa Barbara Co., CA, USA	34.3878	−119.4981
n18	MVZ267040	*B. nigriventris*	N	Ventura Co., CA, USA	34.3512	−119.1474
n19	MVZ267009	*B. nigriventris*	N	Ventura Co., CA, USA	34.3297	−119.1411
n20	MVZ266962	*B. nigriventris*	N	Ventura Co., CA, USA	34.2924	−119.2270
n21	MVZ267031	*B. nigriventris*	N	Ventura Co., CA, USA	34.2790	−119.1406
	MVZ226708	*B. bramei*	Sierran	Tulare Co., CA, USA	35.8342	−118.4411
	MVZ226712	*B. bramei*	Sierran	Kern Co., CA, USA	35.7763	−118.4268
	CAS219746	*B. relictus*	Sierran	Kern Co., CA, USA	35.4786	−118.9589
	MVZ267162	*B. relictus*	Sierran	Kern Co., CA, USA	35.4669	−118.5667
	MVZ220498	*B. simatus*	Sierran	Kern Co., CA, USA	35.5747	−118.5280
	ELJ1922	*B. simatus*	Sierran	Kern Co., CA, USA	35.5669	−118.4013
	MVZ232842	*B. simatus*	Sierran	Kern Co., CA, USA	35.5600	−118.4431

The *B. major* and *B. nigriventris* datasets were analyzed separately following the same methods. Alignment was straightforward as there are no indels in these taxa (see Files S1 and S2). PartitionFinder 1 (Lanfear et al., 2012) was used to test the distinctiveness of three candidate partitions, corresponding to sites at the first, second, and third codon positions, and to select the model of sequence evolution for each supported partition. The best model available in each analysis package was used in maximum likelihood (ML, in Garli v. 2.0) (Zwickl, 2006) and Bayesian inference, as implemented in MrBayes v. 3.2.4 (Ronquist et al., 2012), to infer phylogeographic structure. ML support was estimated with 1000 bootstrap replicates and Bayesian support was estimated from the posterior distribution. In MrBayes, branch lengths and topology were linked across partitions; the prset ratepr=variable option was used to estimate relative rates of the partitions. Other model parameters were unlinked across partitions. For tree and branch length priors, the gamma Dirichlet distribution was used with default parameter values, because branch lengths are more accurately inferred with this prior (Zhang, Rannala & Yang, 2012). Analyses were run for 10 million generations, with the first 1 million discarded as burn-in. The resulting phylogenetic trees were used to assess the origins of the San Joaquin Valley samples.

Formally, the clade including southern *B. major* and *B. pacificus* was treated as the outgroup in analyses of northern *B. major*, because analyses of mtDNA place this clade as the sister taxon to the remaining samples with high confidence (Jockusch & Wake, 2002; Martínez-Solano et al., 2012). The inclusion of more distant outgroups can alter ingroup relationships; thus, we repeated the ML and Bayesian analyses, including model selection, on taxon sets that sequentially pruned clades that fell outside of northern *B. major* (southern *B. major* + *B. pacificus*, then *B. incognitus*, then *B. minor* + *B.* sp.). Similarly, the northern *B. nigriventris* clade was treated as the outgroup for the *B. nigriventris* dataset based on our prior results (Jockusch & Wake, 2002; Jockusch et al., 2012).

### Comparative analysis of divergence levels and isolation-by-distance within southern California

We compared sequence divergence levels and patterns of isolation by distance in *B. major* and *B. nigriventris* to those of four other salamander species in southern California with overlapping ranges: *Aneides lugubris* (Reilly, Corl & Wake, 2015); *Ensatina eschscholtzii eschscholtzii* (Kuchta, Parks & Wake, 2009); *E. e. klauberi* (Devitt et al., 2013); and *Taricha torosa* (Tan & Wake, 1995; Kuchta & Tan, 2006). Mitochondrial sequence data for southern California samples of these taxa (Fig. S1) and their immediate outgroup(s) were obtained from GenBank (Table S3). Sequence divergences for each dataset were estimated in two ways: using the dist.dna function in the R package ape v. 5.2 (Paradis & Schliep, 2019) with the K80 model, and using the FastDist function of the R package ape on ML trees inferred following the methods used for *Batrachoseps* (Fig. S2). A conversion factor of 1.41, calculated from the relative rates of Mueller (2006), was used to standardize *nd4* distances (used for *E. e. klauberi*) to *cytb* distances (used for all other taxa). We tested for a correlation between genetic and geographic distance (the pattern expected under isolation by distance) in each taxon using Mantel tests, as implemented in the R package vegan v.2.5-2 (Oksanen et al., 2018), with 9,999 permutations to estimate significance. To enable comparisons across taxa, we estimated 95% confidence intervals for the relationships between genetic and geographic distance using 1,000 bootstrap replicates; these replicates were composed of independent population pairs (Bohonak, 2002) which circumvents the statistical challenges resulting from non-independence in distance matrices. We also tested whether correlations could be due to simple geographic substructure rather than a broader pattern of isolation-by-distance by repeating our analyses on the largest subclade restricted to mainland southern California in each taxon (Fig. S2). Further details about the analysis methods are provided in File S4.

## Results

### Mitochondrial phylogeography of northern *B. major*

The 78 individuals of northern *B. major* produced 50 unique haplotypes. These were analyzed along with 18 additional haplotypes that were selected to represent the diversity of southern *B. major* and the other taxa nested within *B. major*. Variation occurred at 236 of 784 sites across the full dataset, and at 110 sites (63 of which were parsimony informative) across the northern *B. major* samples. Divergence between the *cytb* clades of northern and southern *B. major* exceeded 9% (K80 distance), while the deepest divergences within northern *B. major* exceeded 4% (Table 3).

**Table 3:** Comparative sequence divergence levels for salamanders from southern California and their close relatives. The number sign (#) is the number of individuals sampled from southern California (including Baja California samples); Mono indicates whether the set of samples from southern California is monophyletic (Y) or not (N); Divergence 1 is between the southern California lineage and its sister taxon from outside of southern California; Divergence 2 is across the basal split within the southern California clade. Divergences are given as average ± SD of the pairwise K80 distances. *B. major* overlap identifies the clades of northern (numbered) and southern (S) *B. major* that each taxon occurs in sympatry or close vicinity to.

**Taxon**	**#**	**Mono**	**Divergence 1**	**Divergence 2**	***B. major*overlap**	
*B. major*	90	N	—	9.0% ±0.8%	N/A	
*B. major* (N)	78	Y	6.5% ± 0.5%	2.8% ± 0.4%^b^ 4.1% ± 0.5%^b^	N/A	
*B. nigriventris* (S)	35^a^	Y	6.7% ± 0.8%	3.4% ± 0.4%^c^ 3.5% ± 0.6%^c^	4, 5	
*E. e. klauberi*	64	Y	9.4% ± 0.3%^d^	6.3% ± 2.0%^d^	3, 5–7, S	
*E. e. eschscholtzii*	3	Y	3.5% ± 0.6%	0.9% ± 0.3%	1–8, S	
*A. lugubris*	10	Y	3.1% ± 0.6%	0.2% ± 0.2%	1, 4–8, S	
*T. torosa*	10	N	2.2% ± 0.6%^e^	0.6% ± 0.0%^e^	4–7, S	

**Notes.**

a *B. nigriventris* from the northern Channel Islands are included in this count; 33 of the samples are from the mainland.

b The position of the basal split is not well supported; these means are calculated across the first and second splits within northern *B. major* in the ML tree.

c The first value is for the average divergence between the northern Channel Island samples and the mainland samples; the second value is the average divergence across the basal node within the mainland samples.

d To facilitate comparisons, the *nd4* divergences have been converted to expected divergences at *cytb*.

e These values were calculated for the more divergent lineage within southern California, which also has deeper within-clade divergences.

PartitionFinder supported the use of separate models for the 1st, 2nd, and 3rd codon positions with all outgroup sets. 2nd and 3rd codon positions were modeled under HKY + I and TrN + G (GTR + G for MrBayes, which has a more restrictive model set), respectively, for all *B. major* taxon sets. 1st codon position models were sensitive to the scope of taxon sampling. TrNef + I + G (K80 + I + G for MrBayes) was favored when all outgroups were included. As outgroups were pruned from the dataset, models with fewer parameters were favored: when the southern *B. major* lineage was excluded, TrNef + I became the preferred model (K80 + I for MrBayes); and when *B. incognitus* was also excluded, K80 + I was the best fitting model. Relationships within the northern *B. major* lineage were relatively unaffected by these changes in models and outgroup sampling. Thus we focus on the results that included the complete set of outgroup lineages.

All samples of northern *B. major* form a clade (bootstrap percent (BP) = 89/posterior probability (PP) = 1) that in this analysis is sister (76/0.98) to a clade (89/0.98) including an undescribed species from Santa Barbara Co., along with *B. minor* from San Luis Obispo Co., far to the northwest (Fig. 2A, Fig. S3). The northern *B. major* clade contains eight subclades, among which relationships are not fully resolved. These subclades are differentiated from each other by a minimum average sequence divergence of 1.9%. All have a posterior probability ≥ 0.94; 7 of 8 have bootstrap support ≥ 70%, while the other is 58%. The samples assigned to each of the eight subclades are geographically cohesive, comprising a generally more inland series of three clades and a more coastal series of five clades (Fig. 2C).

**Figure 4:**
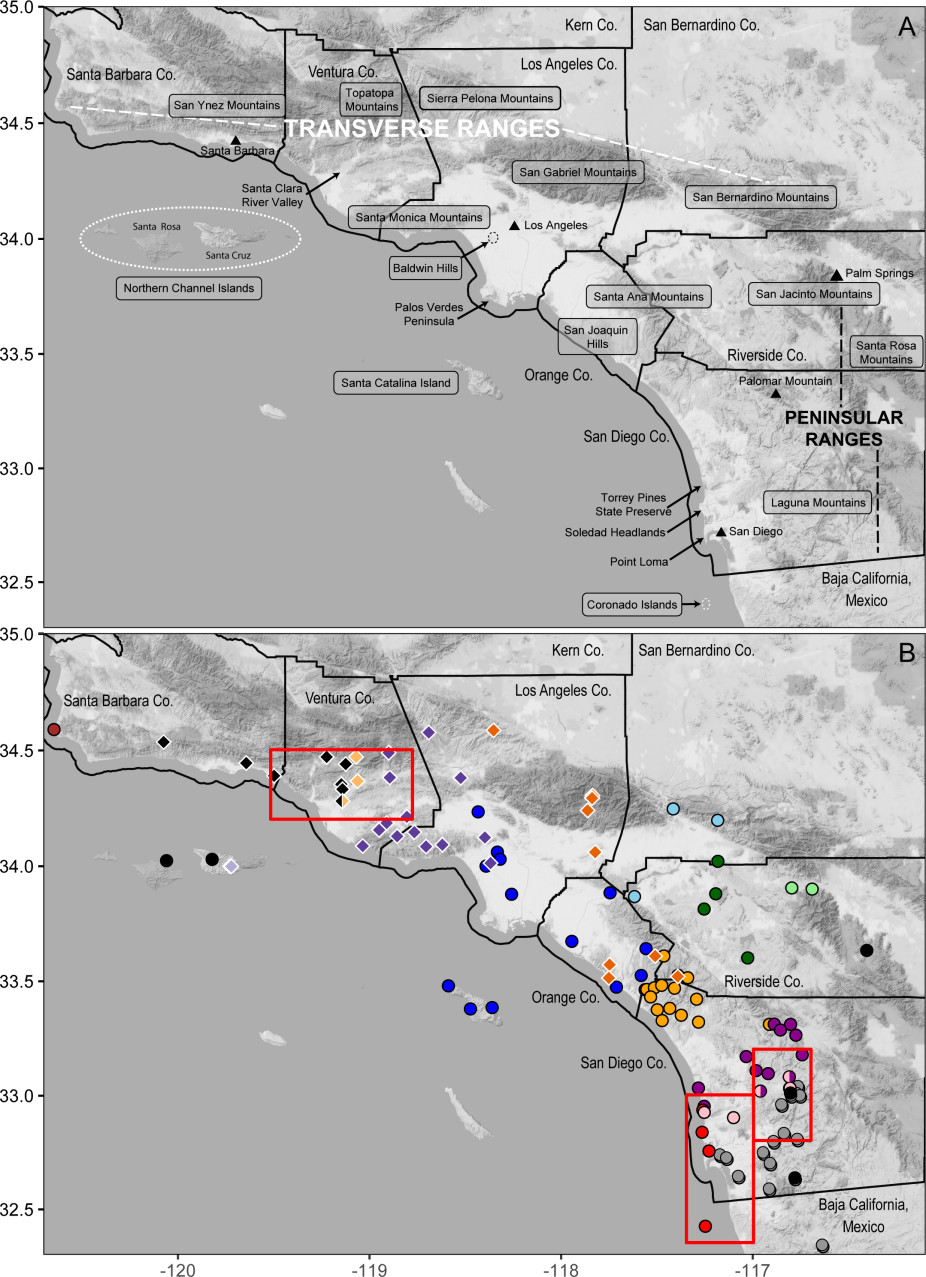
Geographic features in southern California and their correspondence with mitochondrial clades. (A) shaded terrain map naming mountain ranges, islands, other geographic features discussed in the text, counties and cities; (B) *B. major* (circles) and *B. nigriventris* (diamonds) are color-coded by mtDNA lineage as in Figures 2, 3. Red rectangles indicate contact zones that are enlarged in Figure 6 and discussed in more detail in the text. Background map by Stamen Design, used under CC BY 3.0, with map data by OpenStreetMap, under ODbL.

From north to south, the inland clades are distributed as follows (Figs. 2, 4). Clade 1 (light blue) is in the eastern Transverse Ranges, where it extends from the boundary between the San Gabriel and San Bernardino Mountains across the south slopes of the latter. A sample from the eastern edge of the Santa Ana Mountains (pop. 17) was also assigned to Clade 1, although it was excluded from analyses because the data were low quality. Clade 2 (light green) extends along the northeastern edge of the San Jacinto Mountains, on the edge of the desert northwest of Palm Springs. Clade 3 (dark green) is distributed across the Central Perris Block portion of the Peninsular Ranges, south and east of the Santa Ana River, east of the Santa Ana Mountains, west of the San Jacinto Mountains, and north of Palomar Mountain.

Along the coast, the northernmost clade (4, dark blue) is the widest ranging (Figs. 2, 4); it encompasses all Los Angeles Basin samples, as well as samples from the northern Santa Ana Mountains, as far south as Trabuco Canyon, and Santa Catalina Island, one of the southern Channel Islands. Only a single haplotype was found among three individuals from Santa Catalina Island; these individuals are from three widely separated localities on the island. Clade 5 (orange) extends from just north of San Mateo Canyon through the northern Peninsular Ranges as far north as the Ortega Highway where it crosses the crest of the Santa Ana Mountains, and reaches inland as far as Palomar Mountain. Clade 6 (purple) occurs along the coast from just north of the San Dieguito River to Soledad Canyon and it also extends inland to Palomar Mountain. Clade 7 (pink) is narrowly distributed along the southern edge of Clade 6, extending inland as far as the Santa Teresa Valley northeast of Ramona. Along the coast Clade 7 has been found only at the Torrey Pines State Preserve. All five samples of Clade 8 (red) were found within 2 km of the Pacific Ocean, from Torrey Pines State Preserve south of Soledad Canyon at the northern end of its range through the city of La Jolla south to Point Loma and South Coronado Island off the coast of Baja California. Further very fine-scale geographic structuring is observed in several of these clades, with the basal split exceeding 1% divergence in four of them (2, 4, 5, and 7).

The eight northern subclades of *B. major* can be clustered into several more inclusive clades (Fig. 2A, Fig. S3): one uniting the two southern inland clades (Clades 2 and 3; BP = 96/PP = 1) (Fig. 1A) and one uniting all coastal clades except the eastern Los Angeles Basin clade (i.e., Clades 5–8; 51/0.99); within the latter, Clades 7 and 8 are sister taxa (66/0.96). The relationships among the two more inclusive clades (i.e., 5–8, 2–3), the Los Angeles Basin clade (Clade 4), and the Transverse Range clade (Clade 1) are not resolved with confidence by these data.

### San Joaquin Valley samples

Both San Joaquin Valley populations (i1 and i2) were nested within the Los Angeles Basin clade (Clade 4), but within this clade they are not closely related. The sequence from the Hanford individual was unique in the dataset. It differed by <1% (uncorrected p-distance) from five samples from Los Angeles Co. (pops. 1–5), with which it formed the only definite subclade (89/1) within the Los Angeles Basin clade (Fig. 2A, Fig. S3). The *cytb* sequences from the two Bakersfield individuals were identical to one another and to a haplotype found at three localities in Orange Co. (pops. 6–8). Population 7 (Fairview Park, Huntington Beach, Orange Co.) is the locality from which 10 individuals were sampled; it had two haplotypes at approximately equal frequencies (6 and 4); these haplotypes differed at a single nucleotide.

### Mitochondrial phylogeography of southern *B. nigriventris*

For the *B. nigriventris* dataset, PartitionFinder supported the use of TrNef + I (K80 + I for MrBayes), HKY + I and GTR + G for the 1st, 2nd, and 3rd codon positions, respectively. The average sequence divergence between the northern (BP = 100/PP = 1) and southern (75/1) mitochondrial clades within *B. nigriventris* was 8.6% ±0.9%. However, mtDNA from *B. nigriventris* was rendered paraphyletic by a clade comprising the southern Sierran taxa (*B. bramei, B. simatus*, and *B. relictus*; BP = 79; PP = 0.98), which is the sister lineage (BP = 100; PP = 1) to the southern *B. nigriventris* mtDNA lineage (Fig. 3, Fig. S4). The average divergence between these sister clades is 6.7% ± 0.8% (Table 2).

Within the southern *B. nigriventris* lineage, there are four clearly differentiated subclades (BP ≥ 85; PP =1; Fig. 3A, Fig. S4). The average pairwise divergence among these four clades ranges from 3.0–3.5% (with SDs of 0.2–0.7%) (Table 3). This is only slightly shallower than the deepest divergences within northern *B. major*. The deepest split is between an island subclade (light purple; 94/1) restricted to Santa Cruz Island (the only one of the northern Channel Islands that harbors *B. nigriventris*) and a mainland clade (67/0.99), which is in turn subdivided into three geographically cohesive subclades (Fig. 3, Fig. S4). All three have their northern range boundary in the Transverse Ranges. The westernmost of these (light orange; 85/1) appears to have a narrow distribution in the western Transverse Ranges; it has been found along the northern side of the Santa Clara River Valley and a major side drainage, Santa Paula Canyon. It closely approaches the northern *B. nigriventris* mtDNA lineage along its western boundary, and the two have been found in sympatry at Saticoy (pop. 5/n21). On its eastern edge, it is abutted by the central mainland clade (dark purple; 90/1), which is more widely distributed in the western Transverse Ranges, including in the Santa Monica Mountains and Baldwin Hills. The easternmost mainland clade (dark orange; 85/1) makes an inland arc from the Sierra Pelona Mountains across the San Gabriels and then into the Peninsular Ranges, where it occurs in the Santa Ana Mountains and reaches the coast in the adjoining San Joaquin Hills. Both the central and eastern mainland subclades display additional geographic substructure that corresponds well to geology (Fig. 4). Relationships among the three mainland clades are not resolved with confidence (Fig. 3A, Fig. S4).

### Comparative datasets

**Figure 5:**
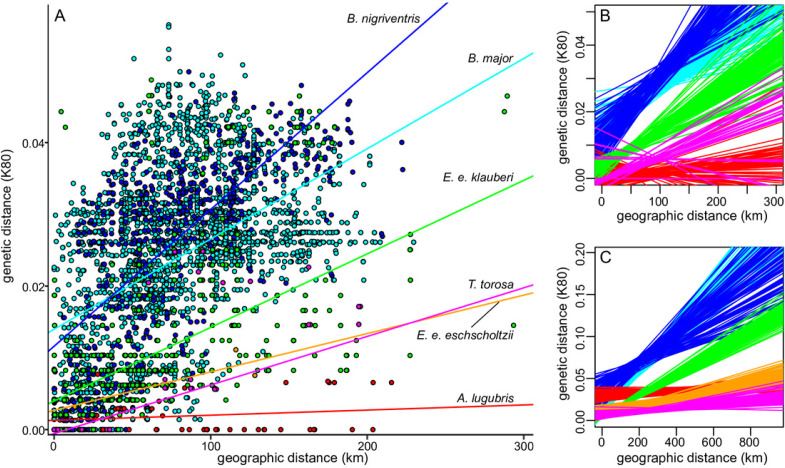
Relationship between genetic and geographic distances. (A) Genetic (K80) versus geographic distance for the southern California samples of each lineage; lines of best fit are based on estimates from the largest clade within southern California. (B–C) The relationship between genetic and geographic distance differs significantly across taxa. Lines of best fit from 100 (of 1000) bootstrap resamplings for each taxon show uncertainty. B shows estimates based on the largest southern California clade (omitted for *E. e. eschscholtzii* because only three populations from the region were sampled) and C shows estimates based on the entire range of the taxon. Species are color-coded as in A.

Levels of sequence divergence within salamander lineages in southern California and between those lineages and their closest relatives outside of southern California vary widely (Table 3). A general pattern of increasing genetic distance with increasing geographic distance was apparent in all taxa (Fig. 5, Fig. S5), as expected under isolation by distance and Mantel tests were significant across the range of each named taxon (Table 3). (Results showed the same general patterns for both distance measures, and we report results from the K80 distances.) In both species of *Batrachoseps* and in *Ensatina e. klauberi*, the relationship between genetic and geographic distance remained significant when the analyses were restricted to the largest monophyletic group within southern California (i.e., northern *B. major*, southern *B. nigriventris*, and the ‘widespread’ clade of *E. e. klauberi*), suggesting that it is due to more general patterns of isolation by distance (IBD), rather than simple geographic substructure. According to the Mantel test, isolation by distance was not detected among the southern California populations of *A. lugubris* and was marginally significant in *T. torosa* from southern California (Table 4), both of which have very incomplete sampling in the region. The best fitting lines of genetic versus geographic distance had relatively steep slopes for *B. major*, *B. nigriventris*, and *E. e. klauberi* (Figs. 5B, 5C). The 95% confidence intervals for the slopes are entirely or almost entirely non-overlapping with those for *E. e. eschscholtzii*, *A. lugubris*, and *T. torosa* in the full dataset, indicating significant differences (Table 4). *Batrachoseps major* and *B. nigriventris* both also differed significantly from *E. e. klauberi*, *B. major* because of a significantly steeper slope and *B. nigriventris* because of a higher intercept. These general patterns held when analyses were restricted to the largest southern California clade sampled from each taxon, although confidence intervals were large for the taxa with small sample sizes (Fig. 5C).

**Table 4:** Isolation-by-distance results. Mantel’s R is the correlation calculated from the Mantel test; ***indicates *p* < 0.005; slope is the estimated slope of the best fit line for genetic (K80) versus geographic (in km) distance, with confidence intervals (CI) estimated using a pairwise bootstrap resampling approach. Results are shown for all samples of a taxon and for the largest monophyletic group restricted to southern California. The sample size for *E. e.eschscholtzii* from southern California was too small for analysis.

	**All samples**		**southern California clade**	
	**Mantel’s R**	slope * 10^4^(95% CI)	**Mantel’s R**	slope * 10^4^(95% CI)
*B. major*	0.67***	2.420 (1.670–3.174)	0.47***	1.216 (0.540–1.920)
*B. nigriventris*	0.43***	1.622 (0.597–2.755)	0.71***	1.898 (1.029–2.685)
*A. lugubris*	0.18***	0.110 (−0.049–0.310)	0.16 (*p* = 0.21)	0.071 (−0.787–0.488)
*E. e. klauberi*	0.82***	1.250 (1.067–1.491)	0.58***	1.025 (0.606–1.508)
*E. e. eschscholtzii*	0.82***	0.435 (0.249–0.670)		
*T. torosa*	0.37***	0.183 (−0.002–0.374)	0.92 (*p* = 0.03)	0.677 (−0.431–1.001)

## Discussion

### Diversification of *Batrachoseps* in southern California

*Batrachoseps major* is one of 8 species-level taxa in the *B. pacificus* species complex (Fig. 1). This complex originated in southern California and displays a highly unusual mitochondrial phylogeographic structure that is intimately related to the historical geomorphology of California (Wake, 2006). The division between the ancestors of the northern and southern *B. major* mitochondrial lineages occurred early in the history of the clade. The ancestor of the southern *B. major* clade subsequently diversified at a time when the northern Channel Islands were farther south, giving rise to *B. pacificus.* Two other mitochondrial lineages split from the ancestor of northern *B. major* and spread to the northwest, together with chunks of the Pacific Plate; these gave rise to two of the four central coast taxa, *B. incognitus* and *B. minor,* and an as yet unnamed taxon situated between these two and northern *B. major*. The ancestors of the other two central coast taxa, *B. gavilanensis* and *B. luciae,* relatively far to the northwest, had split from the others associated with earlier plate fragmentation and movements. The combination of mitochondrial (Figs. 2, 3), nuclear (Fig. 1) and biogeographic data provides evidence of the ability of mtDNA to track ancient geological events that have shaped California.

**Figure 6:**
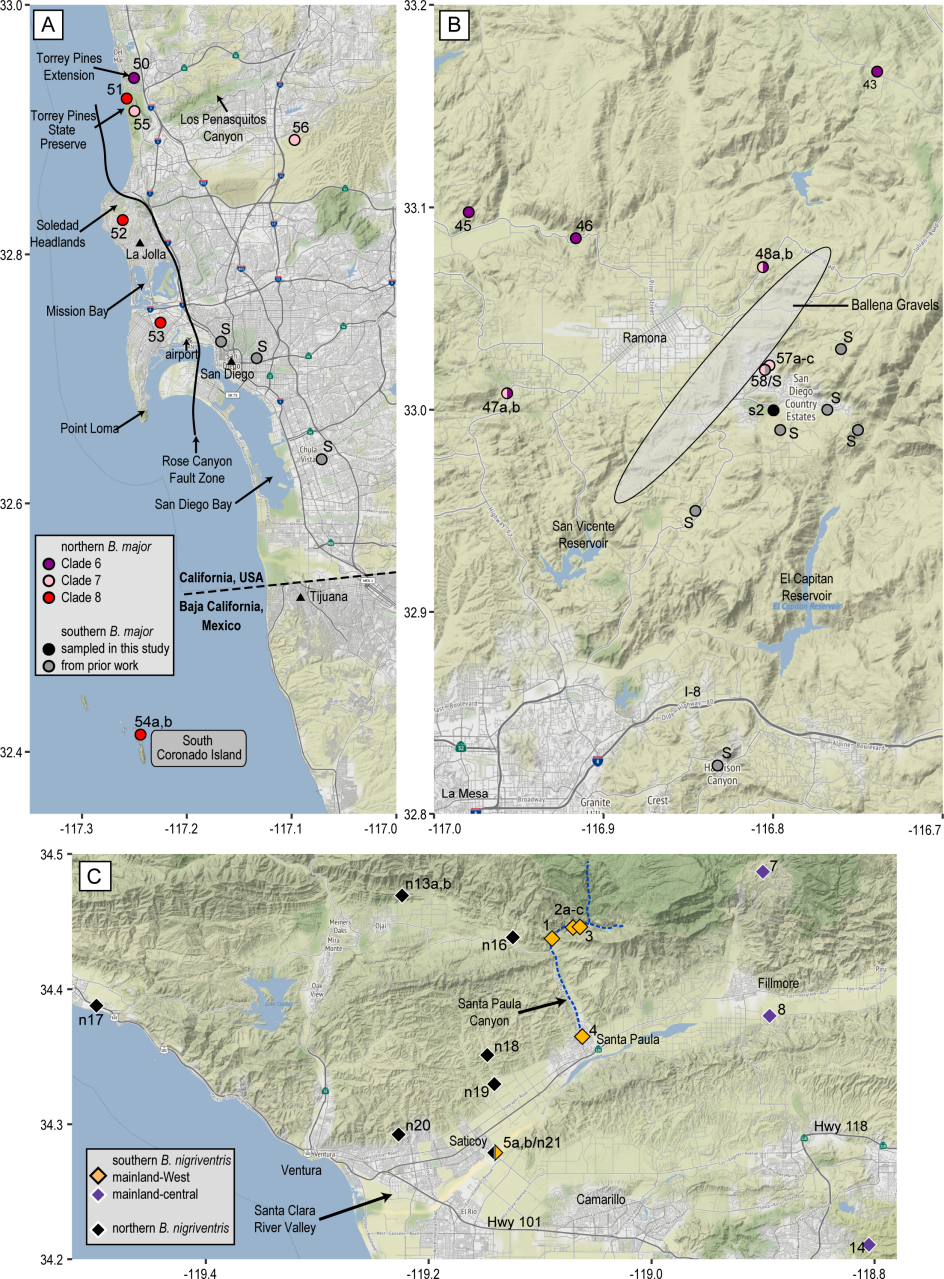
Mitochondrial contact zones. These maps show additional geographic details for three contact zones. Populations are numbered as in Table 1 (*B. major*) and Table 2 (*B. nigriventris*). (A) Coastal San Diego Co. contact zone including Clades 6–8 and southern *B. major*; (B) inland San Diego Co. contact zone involving Clades 6–7 and southern *B. major*; the shaded ellipse shows the approximate region over which the Ballena Gravels are distributed; (C) contact zone between northern and southern *B. nigriventris* mtDNA along the Santa Clara River Valley in Ventura Co. Background maps by Stamen Design, used under CC BY 3.0, with map data by OpenStreetMap, under ODbL; geological features are based on Abbott (1999).

Our new data show that the northern mtDNA lineage of *B. major*, like the southern lineage whose biogeographic structure was investigated in depth by Martínez-Solano et al. (2012), contains multiple mitochondrial clades that are deeply differentiated from each other and highly structured geographically (Fig. 2). The northern lineage is restricted to southern California with the exception of the populations on the Coronado Islands, Mexico (Fig. 4). The distribution of northern *B. major* is to the north and west of that of southern *B. major*, and most of the genetic diversity in the southern lineage is in Baja California. Clades 7 and 8 both contact the southern *B. major* mtDNA lineage, which extends NE to SW from *B. m. aridus* in the Santa Rosa Mountains, through microsympatry between southern *B. major* and Clade 7 inland San Diego Co. (pop. 58 = pop. 25 of Martínez-Solano et al., 2012) (Fig. 6B) in the vicinity of the Ballena Gravels near Ramona, and then through the city of San Diego, where southern *B. major* closely approaches northern Clade 8. The most striking barrier between these northern and southern lineages is the alignment of the North American Monsoon area: the southern lineage occurs where 30–40% of the annual precipitation falls between July and September and the northern lineage is bounded by the area where 20–30% of the annual precipitation falls during this period (see Figure 3 in Metcalfe, Barron & Davies, 2015). One area of near contact between these lineages is the low-lying area between Mission Bay and San Diego Bay bordered to the east by the Rose Canyon Fault Zone, which extends east and then north of Soledad Mountain in La Jolla to enter the sea west of Carmel Mountain. Los Penasquitos Creek and lagoon lie generally within the fault zone, with the larger southern segment of Torrey Pines State Natural Preserve lying to the south and the Torrey Pines Extension lying to the north of the zone. The San Diego Airport essentially spans the fault zone, with southern *B. major* occurring right to its eastern border and northern *B. major* to the west, where it is found in the low western hills leading to Point Loma (Fig. 6A).

The Rose Canyon Fault Zone is also closely associated with the distribution of northern *B. major* Clade 8. Clade 8 occurs in four upland areas along the coast: Torrey Pines State Preserve, the western slopes of Soledad Mountain, Point Loma Heights, and South Coronado Island (Fig. 6A). Point Loma and the Soledad uplift are the only two slivers of mainland still existing west of the Rose Canyon Fault along this portion of the coast (Rockwell, 2010) and the other two localities are associated with northern and southern extensions of this fault system. The entire coastal area from Mission Bay to the vicinity of Tijuana seems likely to have been submerged during the Pliocene (Abbott, 1999; Rockwell, 2010). Perhaps Clade 8 originated at that time from isolates on one of these upland areas such as Soledad Mountain (which today reaches an elevation in excess of 250 m, and likely was land-positive throughout the Pleistocene) (Kennedy & Tan, 2008). Along the coast, Clade 8 occurs within 3 km of clades 6 and 7 spanning the mouth of Soledad Canyon/Carmel Valley, within two parts of Torrey Pines State Preserve (populations 50, 51, and 55). These clade boundaries are also in the immediate vicinity of the Rose Canyon Fault Zone, which doubtless plays an important role in setting the borders of all three clades. The entire Torrey Pines Preserve is only about 8 km^2^ and includes a golf course and extensive beach area so salamander habitat is very limited. It is remarkable that such high mitochondrial diversity occurs locally, with the even more differentiated southern *B. major* relatively nearby.

The range of northern *B. major* is essentially continuous along the coast. Clade 4 is widespread across the Los Angeles Basin and its range matches well the area generally depicted for flood inundation from the 1862 flood of this area (see Figure 1 in Engstrom, 1996) and the area considered to be subjected to Quaternary marine inundation which leads to historic upland habitat fragmentation (see Figure 4A in Vandergast et al., 2006). These two drivers of possible extirpation of the *B. major* populations in this region may explain the low number of unique haplotypes within Clade 4 compared to the other clades within *B. major*. We identified contact between each coastal clade and its geographic neighbors (Fig. 4B). Clades 4 and 5 are in near sympatry in Talega Canyon (populations 11 and 25, separated by ca. 500 m within a continuous patch of trees). Clades 5 and 6 are in near sympatry on the SW slope of Palomar Mountain (populations 38 and 39). In addition to the near sympatry of Clades 6–8 along the coast, Clades 6 and 7 occur in sympatry inland at Woodson Mountain WSW of Ramona (pop. 47) and at Ocean Canyon Ranch (pop. 48) (Fig. 6B). This contact zone is in the region of the Ballena Gravels, which mark the course of an ancient Ballena River (Abbott, 1999), so possibly the adverse rocky, dry soil conditions mark the borders of the different clades and subclades of *Batrachoseps* in the area. The gravels extend from NE to SW of Ramona. Museum samples from the region occupied by the inland clades are much patchier, which may reflect a genuinely patchy distribution. In our limited sampling in the northeastern part of this region, an inland clade most closely approaches a coastal clade on the northeastern end of the Santa Ana Mountains.

Southern *B. nigriventris* overlaps with northern *B. major* on the fringes of the Los Angeles Basin and in the Santa Ana Mountains, as well as the geographically isolated Baldwin Hills within Los Angeles and San Joaquin Hills in Orange County, spanning or approaching three northern *B. major* lineages (Clades 1, 4, and 5; Fig. 4B). It primarily is geographically mutually exclusive with the Clade 4 of *B. major*, where *B. major* is the lowland species and *B. nigriventris* is in the uplands. South and east of the range of *B. nigriventris*, clades of *B. major* take on this higher-elevation niche with the best example being in Palomar Mountain. Southern *B. nigriventris* achieves comparable genetic diversity to the northern lineage of *B. major* across a somewhat more northern range, but without any shared breaks (Fig. 4). The northern and southern *B. nigriventris* mitochondrial lineages meet in the vicinity of the Santa Clara River Valley (Fig. 6C), which formed a deep marine embayment throughout the Pliocene (Hall, 2002). This break has been observed in other taxa, including a flightless beetle (Chatzimanolis & Caterino, 2007), a turtle (Spinks, Thomson & Shaffer, 2010) and a snake (Rodríguez-Robles, Denardo & Staub, 1999). The association of the Northern Channel Islands lineage of *B. nigriventris* (found only on Santa Cruz Island) with samples from the nearby mainland (the rest of the southern lineage) differs from the pattern in the *B. pacificus* group, where *B. pacificus* has much more southern affinities.

### Comparative analysis of divergence in southern California salamanders

Previous comparative phylogeographic analyses of species in the California biodiversity hotspot have identified regions that show concordant patterns of genetic diversity or connectivity across taxa (Rissler et al., 2006; Vandergast et al., 2008). These analyses have focused on diversity adjusted for genetic divergence within a taxon and spatial concordance. Our comparative analyses of divergence and isolation by distance highlight the significantly different timescales across which salamander lineages have diversified in southern California, and the different spatial scales at which this diversity is manifest. There have been at least six independent dispersal events by salamanders into southern California: the plethodontids *B. major* (or its ancestor), southern *B. nigriventris*, *A. lugubris*, and two deeply differentiated lineages within *E. eschscholtzii*, with *E. e. eschscholtzii* likely arriving via a coastal route and *E. e. klauberi* via an inland route (Kuchta, Parks & Wake, 2009; Devitt et al., 2013), and the salamandrid *T. torosa*.

Because the fossil record of plethodontids is poor, few age constraints or calibrations are available, and age estimates are thus determined primarily by the molecular clock calibration used (Kuchta & Tan, 2006; Martínez-Solano et al., 2012; Reilly, Corl & Wake, 2015). Therefore, instead of converting divergences to ages, we compare sequence divergence directly. Given the relatively minor variation in root-to-tip distances for plethodontids in well-modeled mtDNA data (Mueller, 2006), equal divergence depths reflect approximately equal amounts of time within this lineage. Although the duration over which each lineage has persisted in southern California cannot be precisely estimated, the maximum time is generally based on time of divergence from its sister taxon, while the minimum time is generally based on the deepest divergence within southern California.

Among southern California salamanders, our comparative analyses show that northern *B. major*, southern *B. nigriventris*, and *E. e. klauberi* have been diversifying over long periods of time. These three lineages are characterized by both high divergence from their sister taxon outside of southern California and deeply differentiated subclades within southern California (and, in some cases, neighboring areas of Baja California) (Table 3, Fig. S2). The Transverse Ranges separate the southern California ecoregions from more northern mountain ranges. They serve as a major biogeographic disjunction, suggesting that they pose a substantial barrier in many taxa (Calsbeek, Thompson & Richardson, 2003; Lapointe & Rissler, 2005; Rissler et al., 2006; reviewed in Gottscho, 2016). Using the standardly applied molecular clock calibration (of 0.8–1.0% per million years for *cytb*), both the common ancestor of the two *B. major* lineages and *E. e. klauberi* are inferred to have arrived and begun to diversify in southern California and adjoining Baja California well prior to the completed rotation of the Transverse Ranges from their original north-south orientation to their current east–west position (Miller, 2002). The split between northern and southern *B. nigriventris* also predated the completed rotation of the Transverse Ranges. The other three salamanders (*A. lugubris*, *E. e. eschscholtzii*, and *T. torosa*) arrived and diversified much more recently and must have dispersed across the Transverse Ranges. These species generally show lower (or equal) divergences to their sister taxon outside of southern California than the deepest divergences within southern California observed in the early arrivers (Table 3; Fig. S2). These taxa also show low average divergences (<1%) across their basal split within southern California.

In addition to differences in how long they have been present in the region, the southern California salamander lineages differ in the pace at which genetic divergence accumulates over space. This property is dependent on both features of the ecology/geology of southern California and features of the organisms. On large spatial scales, all six lineages show patterns of isolation by distance and geographically cohesive subclades. However, because of differences in the slope of this relationship (Fig. 5, Fig. S5, Table 3), levels of genetic divergence comparable to those observed within southern California in *B. major*, *B. nigriventris*, and *E. e. klauberi* are distributed across much larger geographic distances in the other taxa: across the entire range of *E. e. eschscholtzii* (>600 km, extending from Monterey Bay into northern Baja California), *Aneides lugubris* (>1,000 km, from Humboldt Co. in northern California into Baja California), and *Taricha torosa* (>900 km, from Mendocino Co. in northern California to San Diego Co.). This contrast between the early and late arrivers is maintained when analyses are restricted to the largest sampled monophyletic group within southern California for each lineage*.*

### Factors affecting population persistence

First impressions suggest that southern California offers inhospitable habitat for salamanders, especially those that do not use aquatic retreats. Average annual precipitation in low-lying areas (below about 500 m) south of the Transverse Ranges ranges from 250–550 mm, with rain typically falling on only 30–45 days per year. Additionally, precipitation is very unevenly distributed seasonally, and salamanders must endure six to seven months during the hottest months without any precipitation. Year-to-year variation is high and multiyear droughts are common. On the whole, it is surprising relative to typical salamander habitats elsewhere in North America that terrestrial salamanders are as widespread as they are in the region. Historically, however, the climate was wetter (Robert, 2004; Antinao & McDonald, 2013), with xerification occurring over the last 20,000 years (Kirby et al., 2013).

The ability of salamander species to persist in the region is dependent on access to suitable microsites, including those required for reproduction. All plethodontids are lungless with gas exchange instead occurring across their highly permeable skin. As a consequence, they are very sensitive to desiccation; *B. attenuatus* was found to lose the ability to right itself after an average of only 65 min. in a desiccating environment (Ray, 1958). Thus, persistence requires continuous year-round access to moist microsites. A dry environment also affects fitness in other ways: a recent study documented a large decrease in body condition in *T. torosa* in southern California during the extended drought of 2012–2016 (Bucciarelli et al., 2020). Like *Batrachoseps*, the other plethodontid species have a fully terrestrial life cycle. Because these species develop directly, they do not require access to surface water for reproduction and breeding migrations have not been documented. These two reproductive traits are expected to facilitate persistence. Eggs or other evidence of reproduction have only rarely been observed (Grant, 1958), suggesting that oviposition normally takes place in inaccessible locations. For *Batrachoseps* and *Ensatina*, this is believed to be in subterranean retreats, whereas for *Aneides* it is more likely in tree cavities (Miller, 1944; Stebbins, 1945; Jockusch & Mahoney, 1997; Olson et al., 2006)—all habitat types that are widely distributed. Thus, long-term persistence of *Batrachoseps* at a locality may require little more than land-positive terrain that offers opportunities for dry season underground retreats. In contrast to the plethodontids, *T. torosa* retains a typical salamandrid biphasic life cycle, requiring access to specialized breeding habitats. Models of its population dynamic during extreme drought predict population failures tied to breeding habitats in southern California (Jones et al., 2017).

Signals of long-term persistence, such as the steep isolation-by-distance slopes observed in *Batrachoseps*, also are expected to be stronger with low population connectivity, because otherwise, the signals will be overwritten by gene flow. The fine-scale genetic divergence in *B. major* and *B. nigriventris* likely reflects the low movement levels documented for several species of *Batrachoseps* (Hendrickson, 1954; Cunningham, 1960) in comparison to *Ensatina* (Staub, Brown & Wake, 1995) and *Taricha* (Kuchta, 2005). These patterns of fine-scale geographic structure are reminiscent of those previously described for *B. attenuatus*, the most widely distributed species of *Batrachoseps*, which also occupies a largely continuous, ecologically diverse range (Martínez-Solano, Jockusch & Wake, 2007), and for southern *B. major* (Martínez-Solano et al., 2012). The use of widespread terrestrial breeding localities likely reduces connectivity in *Batrachoseps* and the other three plethodontid lineages. By contrast, *T. torosa* undertakes long-distance movements to and from breeding sites that may exceed 3 km (Kuchta, 2005); these features are expected to increase spatial connectivity and thus decrease the strength of isolation by distance. The contrast in patterns between the two subspecies of *Ensatina* is likely attributable in part to their very different habitats; *E. e. klauberi* occurs at high elevations in habitat that has been subjected to repeated cycles of fragmentation (Devitt et al., 2013) while *E. e. eschscholtzii* is more continuously distributed in lower elevation habitats.

### Introductions

The establishment of vertebrate species outside their native ranges is a growing and, in some cases, ecologically problematic trend (Kraus, 2009; Fitzpatrick et al., 2010). This phenomenon is particularly prevalent among herpetofauna, though relatively rare for caudates (Kraus, 2009). Of 21 species of salamanders that are known or suspected to have become established beyond their natural range, about 50% (11 species) are members of the Plethodontidae, the most speciose clade of extant salamanders (487 of 754 described species, or ∼65%) (AmphibiaWeb, 2020). Our results support two independent introductions of *B. major* to the San Joaquin Valley. These introductions were likely mediated by the nursery trade and facilitated by the same traits that contribute to the extreme build-up of genetic divergence in *Batrachoseps*, especially its ability to persist in small patches of habitat, such as gardens with subsidized moisture. These discoveries add to the growing evidence of introductions of salamander populations (Kraus, 2009).

The mitochondrial phylogeny confirms the identity of samples from the San Joaquin Valley as *B. major* (Fig. 2, Fig. S3). However, the two populations (i1, from Hanford, Kings Co., and i2, from Bakersfield, Kern Co.) appear to have distinct origins within the Los Angeles Basin clade (Clade 4). The Kings Co. sample is nested within Los Angeles Co. samples and the Kern Co. sample is identical to samples from multiple localities in Orange Co. The Orange Co. localities, although geographically cohesive, were separated from each other by 30–36 km; this range exceeds that of all but a few *cytb* haplotypes in *Batrachoseps* (e.g., Martínez-Solano, Jockusch & Wake, 2007; Jockusch et al., 2012; Martínez-Solano et al., 2012). One possible explanation for this wider range is that it reflects reinvasion of lowland areas following Quaternary marine incursions or relatively recent basinwide flooding (Engstrom, 1996; Vandergast et al., 2006). However, only one of these three populations (pop. 8) was collected from a relatively natural habitat, whereas the other two (pops. 6–7) were in landscaped areas. Given the potential for introductions, denser sampling including additional non-landscaped areas in Orange Co. might assist in determining the native range of this haplotype.

The distance from the Bakersfield locality to the nearest known naturally occurring *B. major* population is ca. 135 km and the distance to localities with the same haplotype is 198–230 km. The Hanford population is ca. 250 km from the nearest known natural population of *B. major* and 282–307 km from its closest mitochondrial relatives. Each of the introduced populations is geographically closest to populations of *B. gregarius* (Fig. 2B), which occur 12 km northeast of the Bakersfield *B. major* site along the Kern River at the base of the Sierra Nevada foothills. The Hanford site is ca. 50 km west of the nearest *B. gregarius* locality in Tulare County. There are no records for native populations of *Batrachoseps* from the floor of the San Joaquin Valley south of Fresno County. Prior to European settlement, the San Joaquin Valley was a mix of desert and freshwater marshes (Germano et al., 2011), and seemingly lacked habitat for any species of *Batrachoseps*. Nearly all of these native landscapes were subsequently modified for housing, agriculture, or energy production (Germano et al., 2011). Species that do occur naturally here such as the legless, horned, and leopard lizards show significant genetic structure in their populations (Parham & Papenfuss, 2009; Leaché et al., 2009; Richmond et al., 2017), and if the salamanders were native, we would expect similarly high divergence indicating a long history in this landscape.

The occurrence of two separate introductions suggests that *B. major* may have a propensity for introductions. We believe that this propensity results from two attributes of the species: its ability to survive in hostile (hot and dry) climates if subsidized moisture (as in urban gardens) is provided, and its ability to complete its life cycle within very small patches of suitable habitat (Cunningham, 1960). These attributes increase the probabilities of both transport and subsequent survival required for establishment of a new population. A third population of *Batrachoseps* in the Central Valley, *B. attenuatus* from Riverbank along the Stanislaus River, Stanislaus Co. (Fig. 1), also has been identified as possibly being introduced (Martínez-Solano, Jockusch & Wake, 2007) as have two island populations in San Francisco Bay (Martínez-Solano & Lawson, 2009). Recently, we also identified another population from the Central Valley that is likely to have resulted from an introduction. This population from the vicinity of Stockton, San Joaquin Co. was assumed to belong to *B. attenuatus*, as it is within the native range of that species. However, mtDNA identified it as *B. gavilanensis* (GenBank accession number MT547782), most similar to populations from near Santa Cruz, far to the west. These results suggest that additional introductions are likely to have occurred and point to the possibility that some introductions are cryptic because of the general morphological similarity across many species in the genus.

Because *B. major*, like all species of *Batrachoseps*, deposits terrestrial eggs and lacks an aquatic larval stage (Davis, 1952; Grant, 1958), these salamanders are not dependent on surface water and are able to persist and even thrive in very small patches of habitat, such as residential gardens, empty lots, and freeway right-of-ways. With expansion of urban landscaping and agriculture in areas that were formerly xeric, there is a major increase in annual moisture availability now that has been measured as aseasonal flows in creeks resulting from run-off (Zimmerman et al., 2018). Although transport of salamanders or their eggs through nursery plant containers has not been documented, this is a likely means of translocation, as was seen in the dramatic example of the direct-developing frog *Eleutherodactylus coqui*, introduced to Hawaii (Kraus et al., 1999). The drain holes at the bottom of standard nursery containers offer easy access to a moist retreat for these slender salamanders. In turn, these containers have the potential to relocate adult salamanders as well as eggs, as has been seen in the coqui frogs. California leads the U.S. in production of ornamental nursery plants, with >$1 billion in sales for 2015 (CDFA, 2016). Southern California’s mild climate provides year-round growing conditions and has made this region home to some of the largest commercial nursery growing operations in the United States. Plants from here are shipped throughout the southwestern U.S., raising the possibility that much longer distance introductions have occurred.

### Origin of island populations of *B. major*

Our data also raise questions about the origin of some island populations. In particular, the absence of genetic variation across three samples from widely separated localities on Santa Catalina Island (Fig. 4B), combined with high similarity to mainland samples, would be unexpected if *B. major* had an ancient presence on the island. This mountainous island has no history of connection to the mainland, from which it is presently separated by 35 km; it has been land positive at least throughout the Pleistocene (Hall, 2002). Molecular data provide support for other taxa being native to the island, such as unique mitochondrial lineages of a shrew (*Sorex ornatus*) (Maldonado, Vilà & Wayne, 2001) and a Jerusalem cricket (*Stenopalmatus* n. sp.) (Vandergast et al., 2006), and genetic variation in side-blotched lizards (*Uta stansburiana*) (Mahoney, Parks & Fellers, 2003). The only other salamander reported from Catalina Island is the arboreal salamander, *A. lugubris*; this report is based on a single specimen from 1941 from the Middle Ranch area (Hilton, 1945). An intensive three-year survey of the island failed to detect any evidence of this species (Backlin et al., 2005), suggesting that it was introduced with ranching material, but failed to establish a population.

Our data suggest that, although it is considered an island native (Schoenherr, Feldmeth & Emerson, 1999), *B. major* was also introduced to Catalina Island. The earliest documented specimen of *Batrachoseps* from the island was collected from the resort town of Avalon and reported in 1905 (Van Denburgh, 1905). Regular transport of goods to the island took place due to ranching and mining in the 1860s and subsequently due to its development as a resort beginning in the 1880s (Culver Jr, 2004). The Wilmington area of Los Angeles, just SE of the Palos Verdes Peninsula, was the historic port used at this time, and its vicinity should be sampled to see whether *B. major* there carries the Santa Catalina mtDNA haplotype. Additionally, genetic diversity of *Batrachoseps* from more remote natural chaparral habitats on the island in areas unaffected by nursery plantings should also be investigated to see if they have greater genetic divergence from mainland samples.

*Batrachoseps* occurs on all four of the Coronado Islands; our samples come from South Coronado Island (pop. 54, Fig. 6C), the only one with a history of recent habitation (McCain et al., 2019). A sister group relationship between samples from South Coronado Island and Point Loma (pop. 53) is plausible on geological grounds. However, the near identity of haplotypes (99.7%), historic transport connections between these localities, and extended record of prehistoric use of the islands by humans (McCain et al., 2019) raise the question of whether *Batrachoseps* may also have been introduced to this island. Weighing against this hypothesis are the distribution of *Batrachoseps* on the other three Coronado Islands (which were not sampled) and the recent Pleistocene connections of these islands to the mainland (McCain et al., 2019). Additionally there is low floral endemism on the island even though it has the highest floral diversity per unit area of any of the Baja California northern Pacific Islands (Vanderplank, Rebman & Ezcurra, 2018). Thus, the observed shallow divergence is consistent with these island populations being either natural or introduced.

## Conclusions

We show that the Southern California Slender Salamander, *B. major*, has high mitochondrial genetic structuring within a geologically complex area of southern California, indicative of a long history within this landscape. We identify geographic features correlated with some of the deep phylogeographic breaks as well as contact zones between subclades. Only its congener *B. nigriventris* achieves a similar level of fine-scale genetic structure in the region, while one other salamander, *E. e. klauberi*, also shows a long history in southern California and adjacent regions of Baja California, Mexico. We document the introduction of *B. major* at two sites in the San Joaquin Valley, far outside the native range, demonstrate that these originate from separate sources within urban southern California and suggest that the introductions have occurred via the nursery trade. We hypothesize that features of its life history, including limited movement and direct development, confer *Batrachoseps* with both an ability to persist in small habitat patches, facilitating fine-scale divergence over extended periods, and a propensity for successful establishment when introduced to new areas with sufficient moisture.

##  Supplemental Information

10.7717/peerj.9599/supp-1 Supplemental Information 1 Geographic sampling from this and previous studies of salamanders in southern California Species is indicated by both color and shape, as shown in the legend. Background map by Stamen Design, used under CC BY 3.0, with map data by OpenStreetMap, under ODbL.

10.7717/peerj.9599/supp-2 Supplemental Information 2 Maximum likelihood trees for the comparative phylogeography datasets Numbers along branches indicate bootstrap support >50%. All trees are plotted at the same scale, as indicated by the scale bar showing the branch length corresponding to 0.1 substitutions/site (i.e., 10% divergence) to facilitate comparisons across taxa. For *E. e. klauberi*, *nd4* divergences were converted to the *cytb* scale. Gray tip labels indicate samples that were excluded from the whole taxon dataset in IBD analyses; large asterisks indicate the largest clade within southern California, used in the southern California-only analyses; small asterisks in C and F indicate other southern California clades. Tip labels include voucher name and county. Figure S2A shows the same tree as Fig. 2 and Fig. S2B shows the same tree as Fig. 3.

10.7717/peerj.9599/supp-3 Supplemental Information 3 Phylogeographic structure of northern *B. major* Bayesian tree for *B. major* (with full outgroup sampling), with posterior probabilities > 0.5 shown in blue. Sample names include population number, a letter if more than one individual was sampled from the population, museum or tissue voucher and county. See Table 1 for population coordinates and museum abbreviations.

10.7717/peerj.9599/supp-4 Supplemental Information 4 Phylogeographic structure of southern *B. nigriventris* Bayesian tree for *B.nigriventris* (with full outgroup sampling), with posterior probabilities ¿0.5 shown in blue. Sample names include population number, a letter if more than one individual was sampled from the population, museum or tissue voucher and county; for Sierran taxa, species is indicated. See Table 2 for population coordinates.

10.7717/peerj.9599/supp-5 Supplemental Information 5 Relationship between genetic and geographic distances for southern California salamanders Genetic (K80) versus geographic distance for the more comprehensive geographic sampling of each southern California lineage based on the full range of the taxon; species is indicated by both color and shape, as shown in the legend.

10.7717/peerj.9599/supp-6 Supplemental Information 6 *Cytochrome b* sequences of *B. major* in nexus format

10.7717/peerj.9599/supp-7 Supplemental Information 7 *cytochrome b* sequences of *Batrachoseps nigriventris* in nexus format

10.7717/peerj.9599/supp-8 Supplemental Information 8 GenBank accession numbers for all datasets This includes the GenBank numbers for new submissions for which data aren’t yet publicly available.

10.7717/peerj.9599/supp-9 Supplemental Information 9 Additional information on the comparative analyses of isolation by distance

## Additional Information and Declarations

### Field Study Permissions

The following information was supplied relating to field study approvals (i.e., approving body and any reference numbers): Field sampling was conducted under permits issued by the California Department of Fish and Wildlife (State of California, Scientific Collecting Permits SC-838, SC-8535, SC-013377). Additional permission was obtained from the indicated entities for work at the following localities: Lake Perris (California Department of Parks and Recreation), Lake Skinner (Riverside County Parks), Motte Rimrock Preserve (University of California Natural Reserve System), Starr Ranch (National Audubon Society), San Diego Wild Animal Park (Zoological Society of San Diego).

### Data Availability

The following information was supplied regarding data availability: Newly reported *B. major* data (MN736845–MN736898), *B. gavilanensis* data (MT547782), and *B. nigriventris* data (MN736899–MN736949) are available at GenBank. File S3 contains the GenBank accession numbers for all other samples analyzed. File S1 provides the *B. major* dataset and File S2 provides the *B. nigriventris* dataset. The specimen voucher numbers and collection locations are available in Table 1 (*B. major* dataset) and Table 2 (*B. nigriventris* dataset).
