# Supplementary figures and images for: Slender salamanders (genus Batrachoseps) reveal Southern California to be a center for the diversification, persistence, and introduction of salamander lineages
Source: PeerJ. 2020 Aug 14;8:e9599. doi: 10.7717/peerj.9599 (PMC7430267; doi:10.7717/peerj.9599)

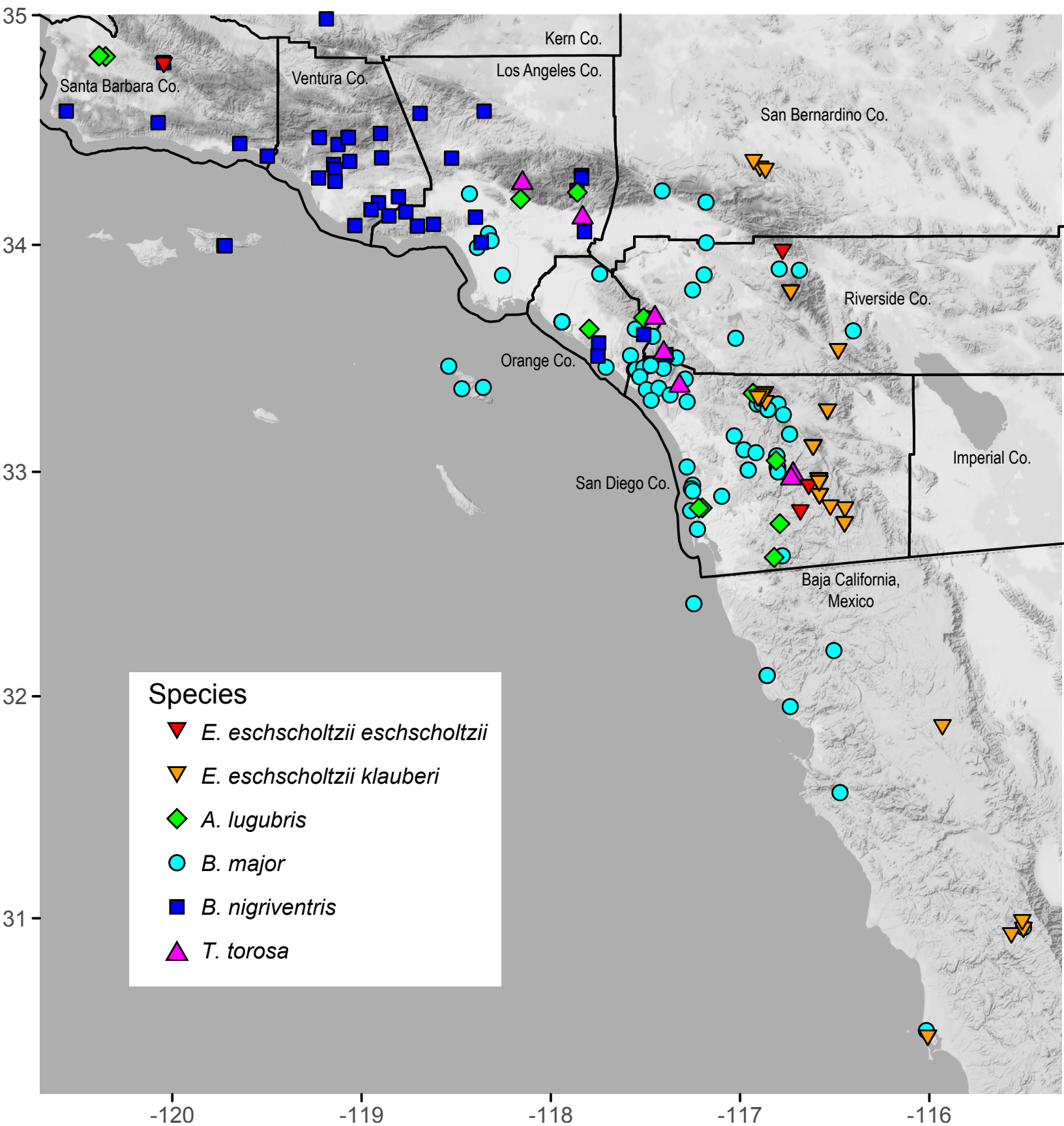

Supplement: Supplemental Information 1 — Species is indicated by both color and shape, as shown in the legend. Background map by Stamen Design, used under CC BY 3.0, with map data by OpenStreetMap, under ODbL. [file peerj-08-9599-s001.pdf]

Bayesian Tree with Posterior Probabilities

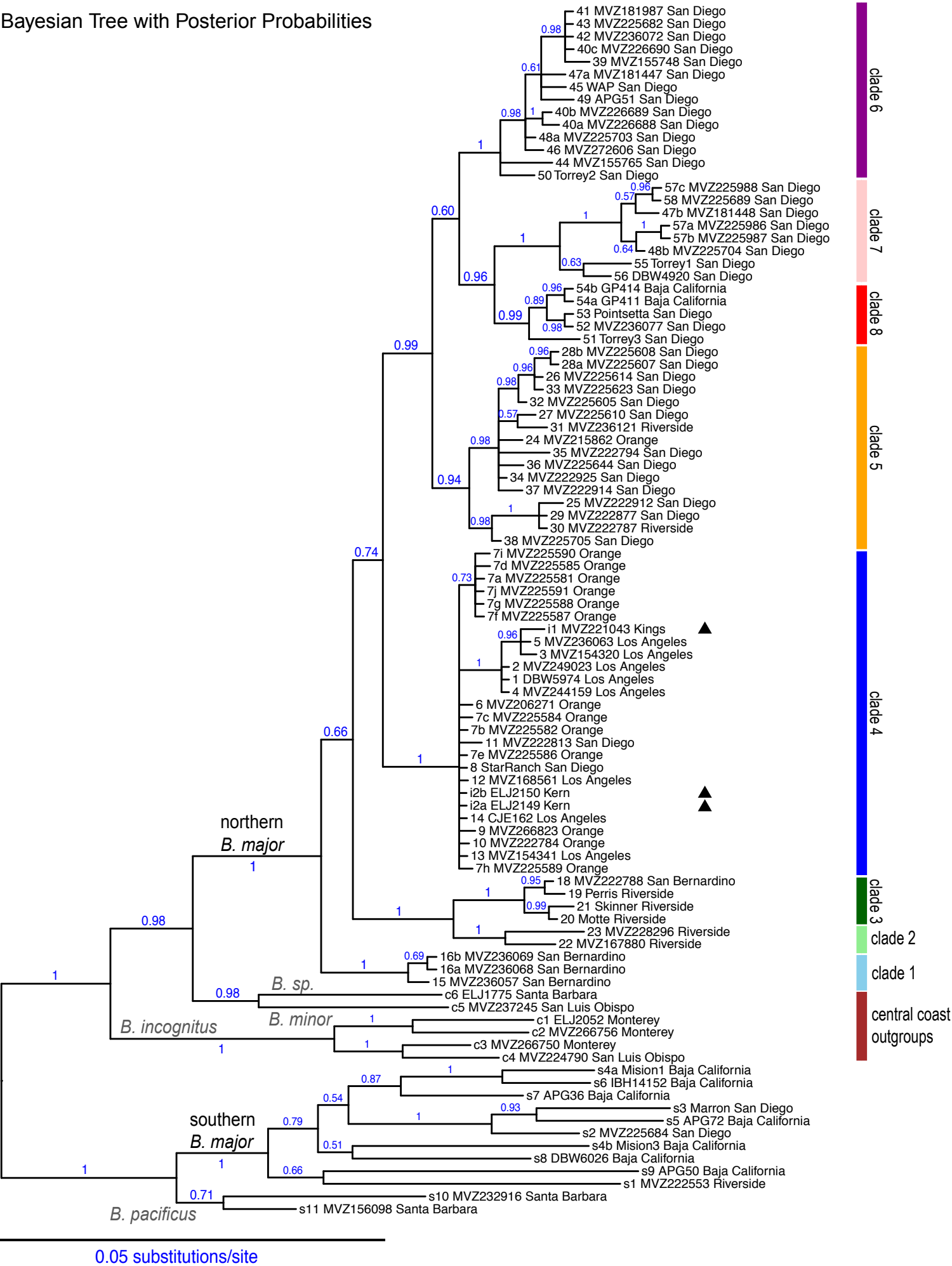

Supplement: Supplemental Information 3 — Bayesian tree for B. major (with full outgroup sampling), with posterior probabilities > 0.5 shown in blue. Sample names include population number, a letter if more than one individual was sampled from the population, museum or tissue voucher and county. See Table 1 for population coordinates and museum abbreviations. [file peerj-08-9599-s003.pdf]

# Bayesian Tree with Posterior Probabilities

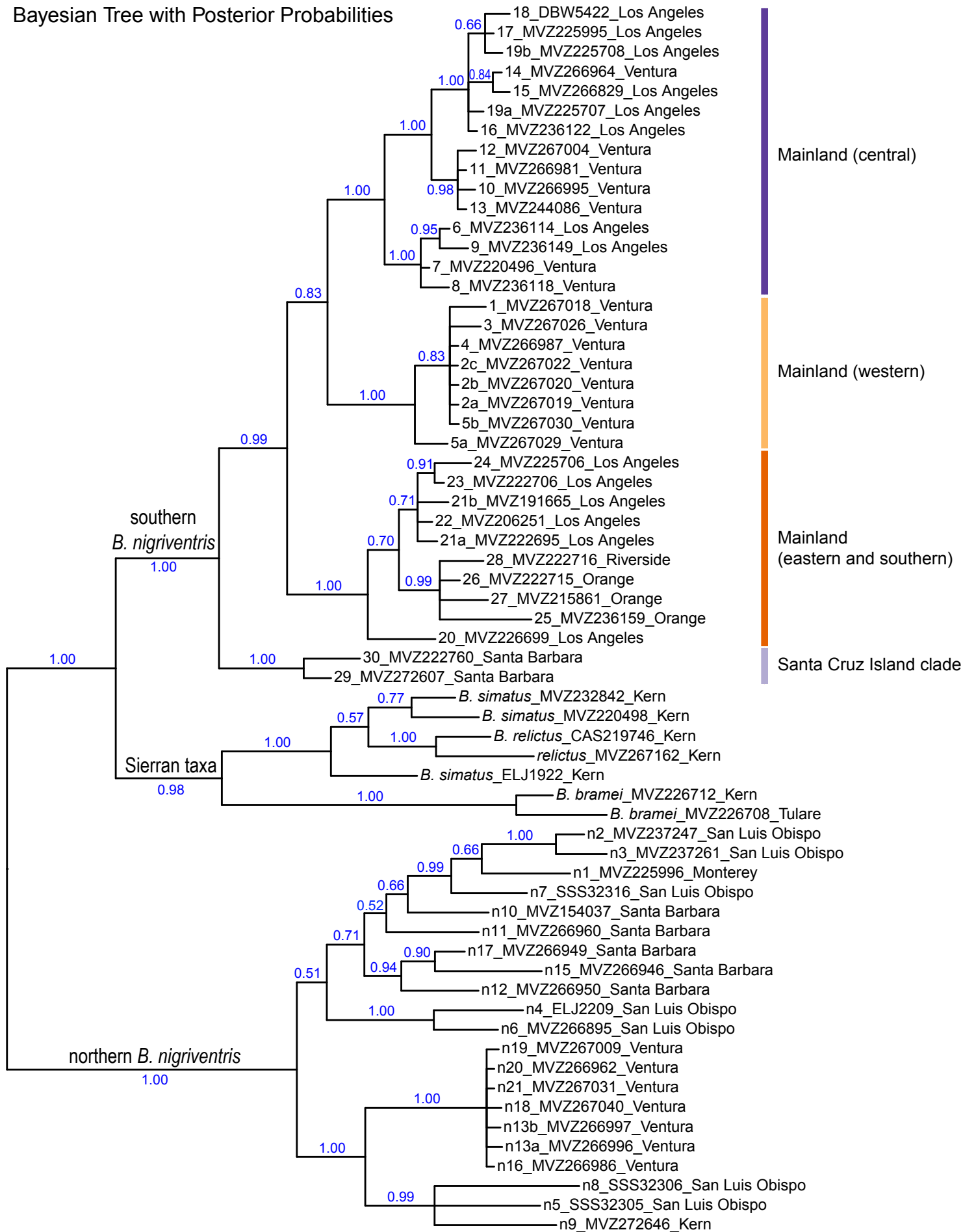

Supplement: Supplemental Information 4 — Bayesian tree for B.nigriventris (with full outgroup sampling), with posterior probabilities ¿0.5 shown in blue. Sample names include population number, a letter if more than one individual was sampled from the population, museum or tissue voucher and county; for Sierran taxa, species is indicated. See Table 2 for population coordinates. [file peerj-08-9599-s004.pdf]

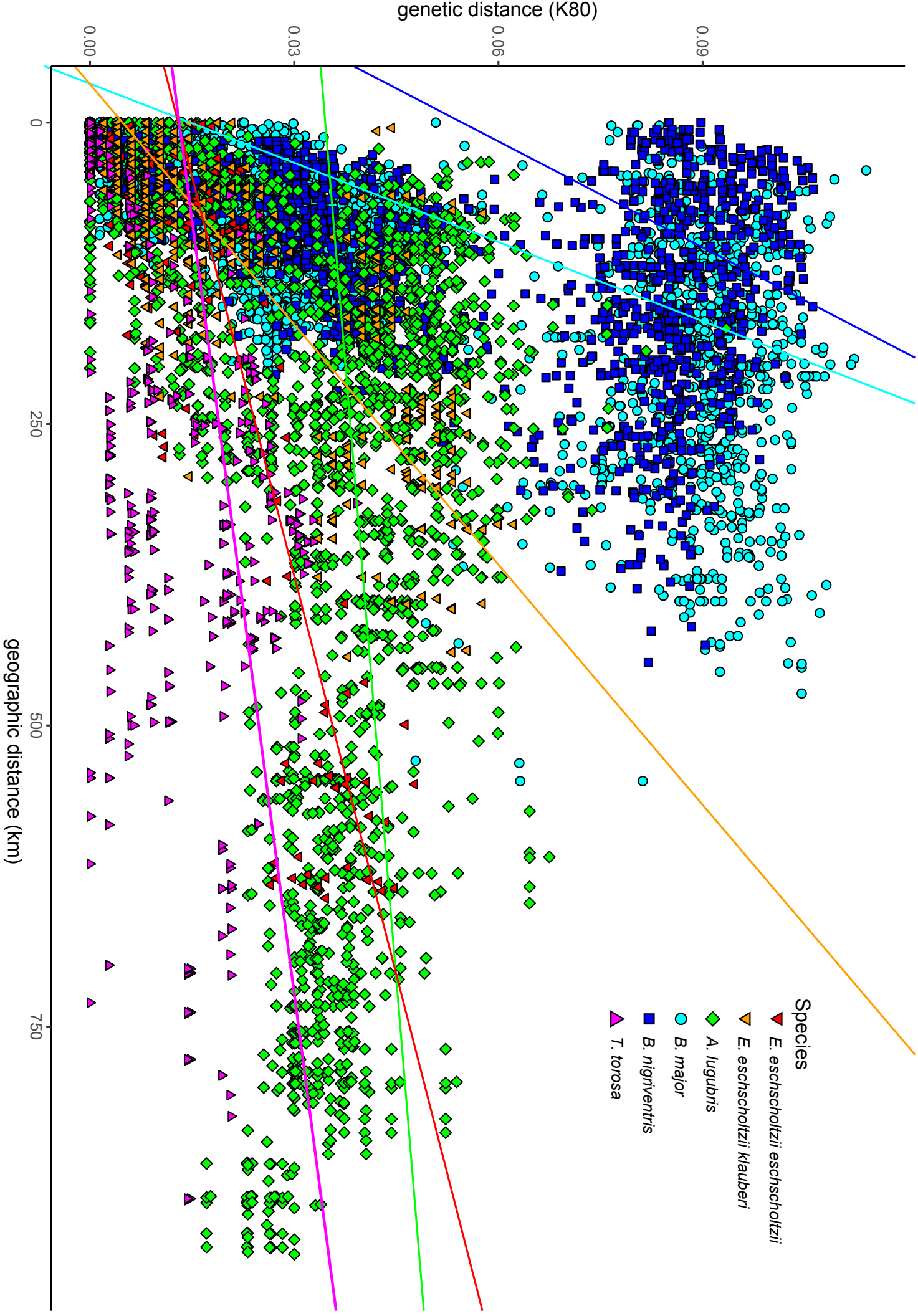

Supplement: Supplemental Information 5 — Genetic (K80) versus geographic distance for the more comprehensive geographic sampling of each southern California lineage based on the full range of the taxon; species is indicated by both color and shape, as shown in the legend. [file peerj-08-9599-s005.pdf]
