# Supplementary material for: Slender salamanders (genus Batrachoseps) reveal Southern California to be a center for the diversification, persistence, and introduction of salamander lineages
Source: PeerJ. 2020 Aug 14;8:e9599. doi: 10.7717/peerj.9599 (PMC7430267; doi:10.7717/peerj.9599)
Supplement: Supplemental Information 9 [file peerj-08-9599-s009.docx]

**Supplemental methods for the comparative analysis of divergence levels and isolation-by-distance**

Each taxon set obtained from GenBank was trimmed to the lineages present in southern California and their immediate outgroup(s), with all southern California samples retained, but sampling reduced in the outgroup when it included many similar or identical sequences. The southern California sample localities from all species are overlaid on Figure S1. All datasets except the *E. e. klauberi* dataset included the same region of *cytb* that was used for *Batrachoseps*. For *E. e. klauberi*, *NADH dehydrogenase subunit 4* (*nd4*) provided much more comprehensive geographic sampling than was available for *cytb*. A conversion factor of 1.41, calculated from the relative rates of Mueller (2006), was used to standardize *nd4* distances relative to *cytb* distances. This conversion factor is conservative from the perspective of testing whether other species accumulate genetic divergence faster than *Batrachoseps*: direct estimate of divergence based on limited *cytb* sampling (N=4 *E. e. klauberi*, 2 *E. e. croceater*) is 8.1 ± 0.4% (Kuchta, Parks & Wake, 2009), whereas the observed *nd4* divergence of 6.7 ± 0.2% converts to 9.4% in *cytb* using the conversion factor from Mueller.

The FastDist function of ape is a tree-based measure of distance (appropriate for a non-recombining region like mtDNA) calculated by summing the branch lengths connecting two terminal taxa. For more divergent samples, it is expected to give a more accurate measure of divergence than K2P. Geographic distances were calculated from latitude and longitude coordinates using the distHaversine function of the R package geosphere v.1.5-7 (Hijmans, 2017).

The Mantel test approach has been criticized on the one hand for having too much power (because other kinds of geographic structure can produce significant results) (Meirmans, 2012) and on the other hand for having too little power (Legendre & Fortin, 2010; Legendre, Fortin & Borcard, 2015); however, they perform relatively well in simulation studies (Diniz-Filho et al., 2013). Given that we found significant IBD, we were not concerned about lack of power. We ensured that strong geographic substructure did not drive the results by repeating these analyses with sampling restricted to the largest monophyletic group restricted to mainland southern California in each taxon except *E. eschscholtzii eschscholtzii*, for which the southern California sample size (n =3) was too low. Following the recommendations of Legendre (Legendre, no date), ordinary least squares regression was used because the data were not normally distributed.

We used the regression of genetic versus geographic distance as an estimate of the strength of the accumulation of genetic distance over space. To estimate confidence intervals for these lines, we adopted the bootstrap sampling approach described in (Bohonak, 2002), which samples (without replacement) genetic and geographic distance values from pairs of populations. Because each population appears in only a single pair, these subsamples circumvent the non-independence inherent in distance matrices, which complicates statistical analyses. As a result, the number of points used to calculate the slope in each bootstrap replicate is half (or half minus 0.5) of the number of populations. 1000 population-pair bootstrap replicates for each dataset (randomly generated in R) were then subject to ordinary least squares regression, and the 95% confidence intervals estimated from these values by trimming the most extreme 2.5% of values on each tail. These analyses were repeated on the southern California data subsets with sufficient sample sizes.

**References**

Bohonak AJ. 2002. IBD (Isolation by Distance): a program for analyses of isolation by distance. *The Journal of Heredity* 93:153–154. DOI: 10.1093/jhered/93.2.153.

Diniz-Filho JAF, Soares TN, Lima JS, Dobrovolski R, Landeiro VL, de Campos Telles MP, Rangel TF, Bini LM. 2013. Mantel test in population genetics. *Genetics and Molecular Biology* 36:475–485. DOI: 10.1590/S1415-47572013000400002.

Hijmans RJ. 2017. *geosphere: Spherical Trigonometry . R package version 1.5-7.*

Kuchta SR, Parks DS, Wake DB. 2009. Pronounced phylogeographic structure on a small spatial scale: geomorphological evolution and lineage history in the salamander ring species *Ensatina eschscholtzii* in central coastal California. *Molecular Phylogenetics and Evolution* 50:240–255. DOI: 10.1016/j.ympev.2008.10.019.

Legendre P. Model II regression user’s guide, R edition. Available at https://cran.r-project.org/web/packages/lmodel2/vignettes/mod2user.pdf

Legendre P, Fortin M-J. 2010. Comparison of the Mantel test and alternative approaches for detecting complex multivariate relationships in the spatial analysis of genetic data. *Molecular Ecology Resources* 10:831–844. DOI: 10.1111/j.1755-0998.2010.02866.x.

Legendre P, Fortin M, Borcard D. 2015. Should the Mantel test be used in spatial analysis? *Methods in Ecology and Evolution* 6:1239–1247. DOI: 10.1111/2041-210X.12425.

Meirmans PG. 2012. The trouble with isolation by distance. *Molecular Ecology* 21:2839–2846. DOI: 10.1111/j.1365-294X.2012.05578.x.

Mueller RL. 2006. Evolutionary rates, divergence dates, and the performance of mitochondrial genes in Bayesian phylogenetic analysis. *Systematic Biology* 55:289–300. DOI: 10.1080/10635150500541672.
