# Supplementary material for: Slender salamanders (genus Batrachoseps) reveal Southern California to be a center for the diversification, persistence, and introduction of salamander lineages
Source: PeerJ. 2020 Aug 14;8:e9599. doi: 10.7717/peerj.9599 (PMC7430267; doi:10.7717/peerj.9599)
Supplement: Supplemental Information 2 — Numbers along branches indicate bootstrap support >50%. All trees are plotted at the same scale, as indicated by the scale bar showing the branch length corresponding to 0.1 substitutions/site (i.e., 10% divergence) to facilitate comparisons across taxa. For E. e. klauberi, nd4 divergences were converted to the cytb scale. Gray tip labels indicate samples that were excluded from the whole taxon dataset in IBD analyses; large asterisks indicate the largest clade within southern California, used in the southern California-only analyses; small asterisks in C and F indicate other southern California clades. Tip labels include voucher name and county. Figure S2A shows the same tree as Fig. 2 and Fig. S2B shows the same tree as Fig. 3. [file peerj-08-9599-s002.pdf]

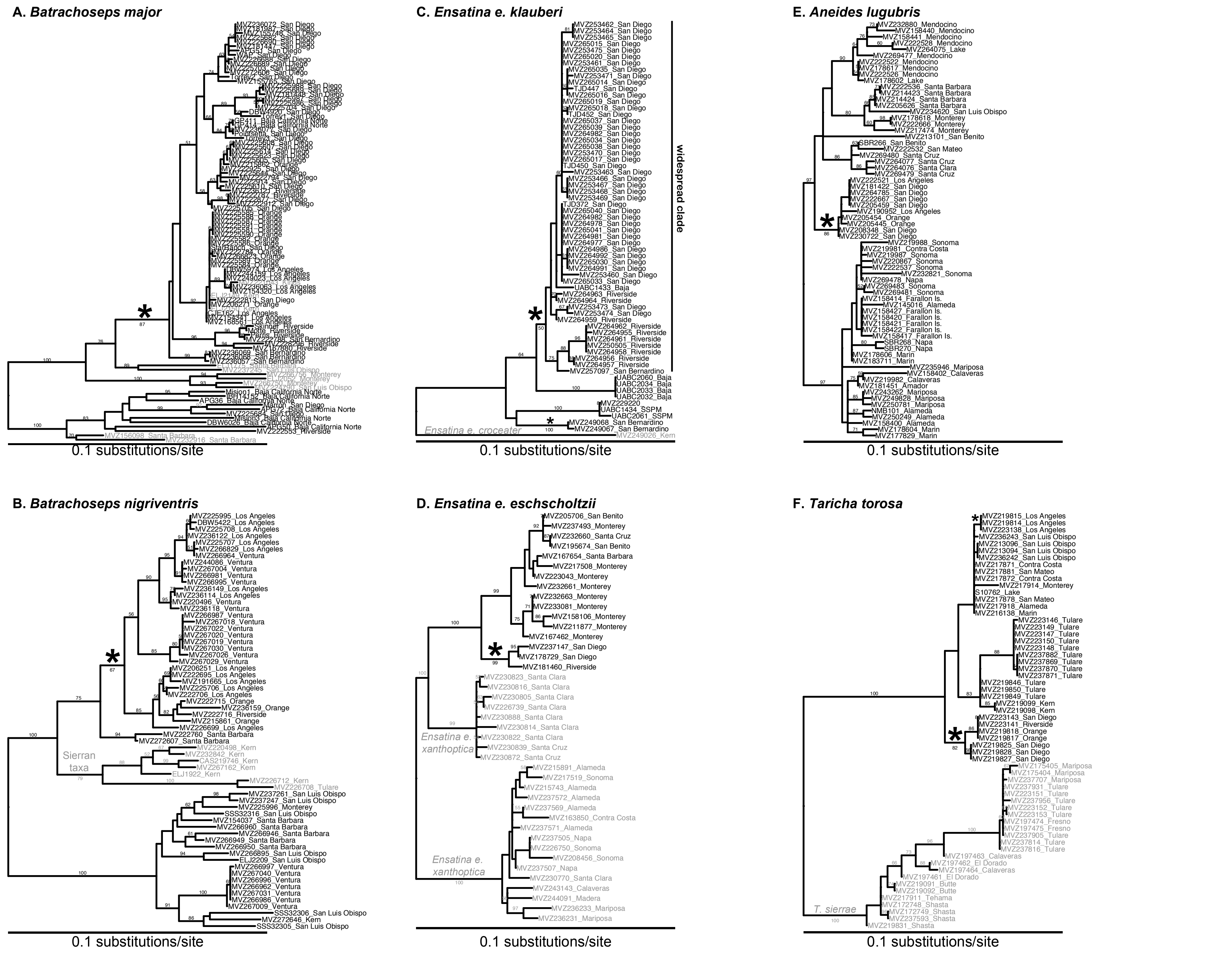
A. Batrachoseps major
0.1 substitutions/site
B. Batrachoseps nigriventris
Sierran taxa
0.1 substitutions/site
C. Ensatina e. klauberi
widespread clade
Ensatina e. croceater
0.1 substitutions/site
D. Ensatina e. eschscholtzii
Ensatina e. xanthoptica
Ensatina e. xanthoptica
0.1 substitutions/site
E. Aneides lugubris
0.1 substitutions/site
F. Taricha torosa
T. sierrae
0.1 substitutions/site
